# Astrocytes Drive Divergent Metabolic Gene Expression in Humans and Chimpanzees

**DOI:** 10.1093/gbe/evad239

**Published:** 2023-12-30

**Authors:** Trisha M Zintel, Jason Pizzollo, Christopher G Claypool, Courtney C Babbitt

**Affiliations:** Department of Biology, University of Massachusetts Amherst, Amherst, MA, USA; Molecular and Cellular Biology Graduate Program, University of Massachusetts Amherst, Amherst, MA, USA; Department of Biology, University of Massachusetts Amherst, Amherst, MA, USA; Molecular and Cellular Biology Graduate Program, University of Massachusetts Amherst, Amherst, MA, USA; Organismic and Evolutionary Biology Graduate Program, University of Massachusetts Amherst, Amherst, MA, USA; Department of Biology, University of Massachusetts Amherst, Amherst, MA, USA; Molecular and Cellular Biology Graduate Program, University of Massachusetts Amherst, Amherst, MA, USA; Organismic and Evolutionary Biology Graduate Program, University of Massachusetts Amherst, Amherst, MA, USA

**Keywords:** astrocytes, neurons, evolution, metabolism, genomics, brain

## Abstract

The human brain utilizes ∼20% of all of the body's metabolic resources, while chimpanzee brains use <10%. Although previous work shows significant differences in metabolic gene expression between the brains of primates, we have yet to fully resolve the contribution of distinct brain cell types. To investigate cell type–specific interspecies differences in brain gene expression, we conducted RNA-seq on neural progenitor cells, neurons, and astrocytes generated from induced pluripotent stem cells from humans and chimpanzees. Interspecies differential expression analyses revealed that twice as many genes exhibit differential expression in astrocytes (12.2% of all genes expressed) than neurons (5.8%). Pathway enrichment analyses determined that astrocytes, rather than neurons, diverged in expression of glucose and lactate transmembrane transport, as well as pyruvate processing and oxidative phosphorylation. These findings suggest that astrocytes may have contributed significantly to the evolution of greater brain glucose metabolism with proximity to humans.

**Significance**

Astrocytes are glial cells that have recently be shown to have important roles in maintenance of synaptic signaling, provisioning of critical metabolites, and the maintenance of blood–brain barrier. We performed RNA-seq on neural progenitor cells, neurons, and astrocytes that we generated from human and chimpanzee induced pluripotent stem cells. We found interspecies differential expression to be twice as high when compared with neurons, suggesting that significant differences in gene expression between human and chimpanzee brains may be due to altered signaling in astrocytes.

## Introduction

Though primates exhibit widespread variation in many phenotypes, including anatomy, behavior, and cognition, the extent of these phenotypic differences is not substantially larger than differences in genome sequence (Varki and Altheide 2005; Varki and Varki 2015). One of those traits that defines primates is a significantly larger brain relative to body size, for which humans exhibit the greatest amount of difference.

Within primates, selective differences in the genome can be linked to diet and metabolism, suggesting selection has optimized different metabolic processes in lineage-dependent ways (Stringer and Andrews 1988; Schaffner et al. 2005; Fagundes et al. 2007; Babbitt et al. 2010; Haygood et al. 2010; Babbitt et al. 2011; Bauernfeind et al. 2015). The human brain is more energetically costly than that of other primates, utilizing ∼20% of all of the body's metabolic resources, in comparison with nonhuman primate brains that use <10% (Mink et al. 1981; Hofman 1983). Importantly, allometry alone does not explain the increase in human brain appropriation of glucose metabolism at this proportion (Martin 1981; Karbowski 2007; Yu et al. 2014). There is evidence that sheer increase in neuron number can explain at least part of the energetic demand of the human brain (Herculano-Houzel 2011). However, interspecies differences in the contribution of metabolism of astrocytes versus neurons to metabolic capacity at the organ level remain largely unexplored.

Many of these changes in brain metabolism have been hypothesized to coincide with other trait changes, particularly those related to shifts in diet known to be important in hominin evolution, such as an increase in meat products, increased quality of food, and agriculture (Brown et al. 1985; McHenry 1992; McHenry 1994; Aiello and Wheeler 1995; Peters 2007; Shea 2007; Babbitt et al. 2011). A recent study investigating an extensive number of primates is consistent with the idea that diet is a better predictor of brain size than social group structure (DeCasien et al. 2017). The expensive-tissue hypothesis posits that a trade-off in energy allocation for the development of a larger, metabolically demanding brain in primates coincided with a reduction in gut tissue (Stearns 1992; Aiello and Wheeler 1995; West et al. 2001; Pontzer et al. 2014). Similarly, an increase in energy-storing tissue (adipose tissue) at the expense of energetically demanding muscle tissue may have also allowed for greater allocation to a larger brain (Leonard and Robertson 1994; Leonard and Robertson 1997; Leonard et al. 2003). There is evidence that the higher metabolic costs of the human brain influences the protracted development of body growth rate (Kuzawa et al. 2014). Evolutionary differences in brain metabolism are a subset of studied differences in metabolic traits that exhibit intriguing differences across primate species. Primates exhibit a lower total energy expenditure (TEE) to body size ratio than nonprimates, and furthermore, humans have greater TEE than closely related great ape species (chimpanzees, bonobos, gorillas, and orangutans) due in large part to increased basal metabolic rate (BMR), all of which is consistent with the reallocation of metabolic investment to the brain (Pontzer et al. 2014, 2016). These in vivo (whole organism) studies further suggest an important link between evolutionary differences in metabolism and the uniqueness of the primate brain.

Similar to organism-level investigations, there is also molecular evidence supporting the evolution of metabolic processes (e.g. oxidative phosphorylation [OXPHOS]) in the primate brain with phylogenetic proximity to humans. Metabolism in the brain is critical for neurological function, as it provides cellular energy and critical biomolecules necessary for the complex cellular network characteristic of the brain (Brown et al. 2001; Tekkök et al. 2005; Nelson et al. 2008; Vander Heiden et al. 2009; Raichle 2010; Vander Heiden et al. 2010; Bauernfeind and Babbitt 2014). Cellular metabolism involves the breakdown of fuel molecules to produce energy or other molecules through multiple interconnected pathways, including glycolysis, OXPHOS, and the pentose phosphate pathway (PPP). Enrichments for metabolic processes in genes and gene regulatory regions undergoing positive selection are a common thread in gene expression analyses from whole primate brain tissue (Haygood et al. 2007; Kosiol et al. 2008; Uddin et al. 2008; Babbitt et al. 2010; Haygood et al. 2010; Bauernfeind et al. 2015). Interestingly, there are lineage-dependent differences in the specific pathways enriched between species (e.g. glucose and carbohydrate metabolism in humans and glycogen and acyl-CoA metabolism in chimpanzees) (Haygood et al. 2007; Kosiol et al. 2008; Uddin et al. 2008). Within anthropoids, genes encoding the subunits of cytochrome c oxidase, the final component of the electron transport chain, show an accelerated rate of evolution in their sequences compared with any other placental mammals (Wu et al. 1997; Grossman et al. 2001; Wildman et al. 2002; Uddin et al. 2008). These molecular changes suggest increased control over the mechanisms that process glucose (Grossman et al. 2001; Goldberg et al. 2003; Grossman et al. 2004; Uddin et al. 2008; Hüttemann et al. 2012). Further understanding gene–phenotype relationships between genetic changes (both in coding and noncoding regulatory portions of the genome) and observed metabolic differences in primates will contribute to a greater understanding of proximate influences on larger evolutionary trends in primates. These findings also highlight a need to investigate not only glucose metabolism and energy production but also that of other macromolecules (e.g. lipids, amino acids, and nucleic acids) for a more comprehensive understanding of differences in cellular metabolism in neural cells of primates.

Many previous comparative primate studies using functional genomics have determined significant differences in expression between humans, chimpanzees, and other primate species (primarily, rhesus macaque) (Khaitovich et al. 2004; Oldham et al. 2006; Blekhman et al. 2008; Babbitt et al. 2010; Konopka et al. 2012; Bakken et al. 2015; Bauernfeind et al. 2015). However, as many investigations of primate evolution and humans in particular often are, these studies have been largely been limited to utilizing posthumous tissue samples, oftentimes opportunistically obtained. Recent advances in induced pluripotent stem cell (iPSC) technology have allowed for the generation of brain organoids as an in vitro model of primate brain development. There have been a number of studies using iPSCs to generate brain organoids as an in vitro model for brain development (Camp et al. 2015; Luo et al. 2016; Amiri et al. 2018; Velasco et al. 2019), including 1 comparative study between humans, chimpanzees, and rhesus macaques (Kanton et al. 2019). The use of iPSC-derived samples has shown great promise in understanding brain development and function in greater detail. While monolayer culturing of iPSC-derived cells does not recapitulate the complexity of the primate brain as well as brain organoids, they are far more feasible in both cost and time and have been used extensively to investigate brain cell type (CT)-specific mechanisms of disease (Zhao et al. 2017; Cho et al. 2019; di Domenico et al. 2019; Penney et al. 2020).

The findings of interspecies divergence in brain metabolism are intriguing; however, a CT-specific comparison would more fully inform our understanding of distinct cellular contributions to interspecific differences in neurological function (Romero et al. 2015). Two of the major CTs in the brain are neurons and astrocytes. Neurons function in neurological processes like cognition and perception largely by transmitting chemical and electrical signals throughout complex cellular networks. However, metabolic programs have been shown to shift as neural progenitor cells (NPCs) differentiate into more mature CTs (Zheng et al. 2016). Nondividing, mature neurons are known to have very little capacity for specific metabolic processes (e.g. glycolysis) and rely on metabolite shuttling from another CT, astrocytes (Almeida et al. 2004; Herrero-Mendez et al. 2009; Sonntag et al. 2017). Astrocytes, despite being the most abundant CT in the central nervous system (Nedergaard et al. 2003), have traditionally been considered support cells for neurons without significant relevance to neural function. However, recent work has determined critical roles of astrocytes in neural function including provisioning of metabolites to neurons for energy (Pellerin and Magistretti 1994; Volkenhoff et al. 2015; Mächler et al. 2016) and enhancing synaptic processes (Meyer-Franke et al. 1995; Diniz et al. 2012). These findings point to a need to characterize the important differences among a variety of CTs, not only in neurons but in other metabolically relevant brain CTs such as astrocytes between species to understand how the primate brain has evolved.

We hypothesize that there are important CT-specific metabolic changes between human and chimpanzee brains and that astrocytes contribute, at least in part, to these differences. To investigate these changes, we established protocols for the differentiation of iPSCs into mature, functional neurons and astrocytes from humans and chimpanzees from multipotent NPCs. This novel comparative approach allowed us to functionally test each CT in the absence of other CTs in a defined, controlled environment. In order to determine adaptive interspecies differences in gene expression and metabolism in a CT-specific manner, we conducted RNA-seq on human and chimpanzee NPCs, neurons, and astrocytes. We determined significant interspecies differential expression (DE) in all 3 CTs with the greatest degree of difference in astrocytes. Pathway enrichments revealed significant differences in gene expression between species across all CTs as well as CT-specific changes of gene expression in glucose and lactate transmembrane transport suggestive of a higher capacity for energetic, rather than biosynthetic, metabolic phenotypes in human astrocytes. This work demonstrates a putative CT-specific mechanism by which astrocytes may have played a role in conferring the adaptive metabolic capacity of the human brain. It also contributes to a growing number of studies demonstrating the importance of considering astrocytes in presumably human-specific phenotypes, including neurodegenerative diseases.

## Results

### RNA-Seq of Human and Chimpanzee iPSC-Derived Neural Cells

**Fig. 1.**
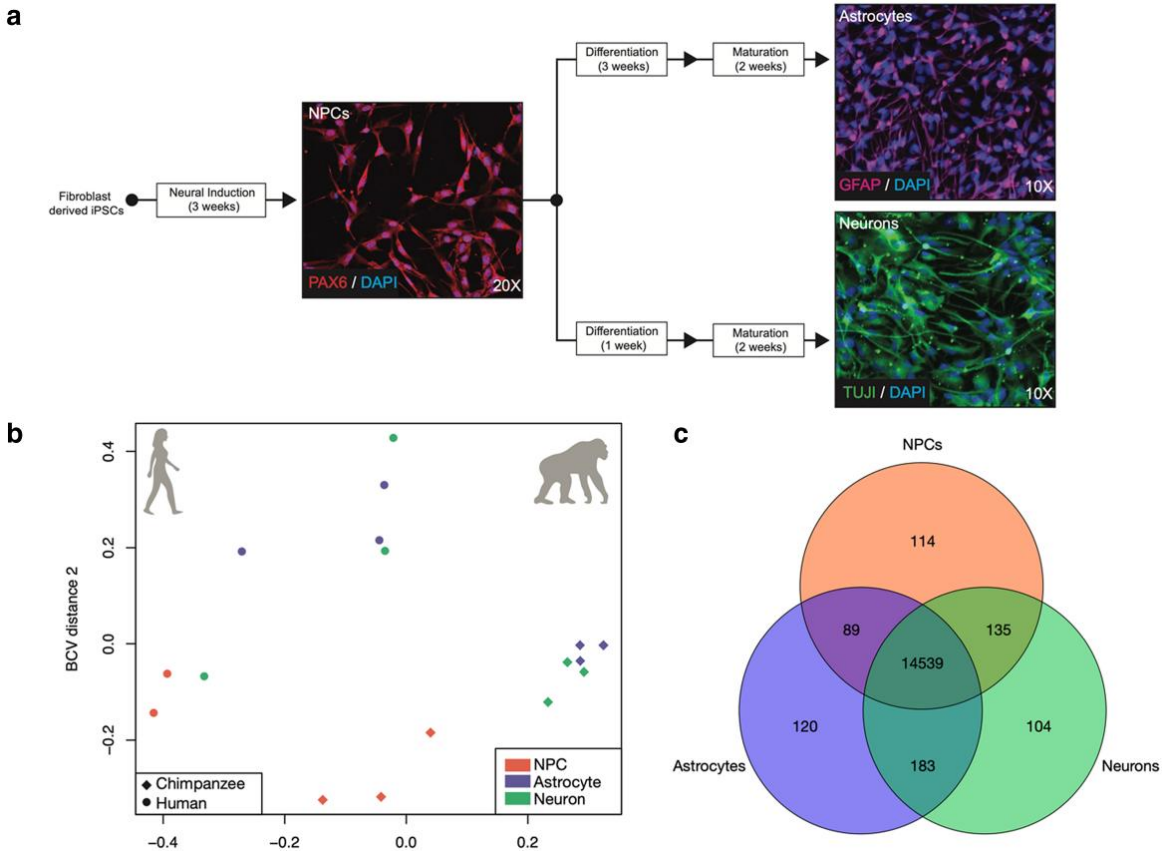
Patterns of gene expression variation of iPSC-derived neural cells from humans and chimpanzees. a) The differentiation schematic and representative immunofluorescent photos of iPSC-derived NPCs stained with PAX6, astrocytes stained with GFAP, and neurons stained with TUJ1. b) A PCoA of the iPSC-derived NPCs, neurons, and astrocytes transcriptomes. c) A Venn diagram of the overlap in expression across CTs. Further details for samples are included in supplementary table S1, Supplementary Material online.

We took a comparative genomics approach to investigating interspecies differences in neural CT-specific gene expression between humans and chimpanzees. Three cell lines per species, representing 3 individuals, were used. These cell lines were originally obtained as fibroblasts from minimally invasive skin biopsies, reprogrammed into iPSCs, and have been validated for their pluripotency and differentiation abilities (Romero et al. 2015; Burrows et al. 2016; Blake et al. 2018; Pavlovic et al. 2018; Ward et al. 2018; Eres et al. 2019; Ward and Gilad 2019). iPSCs from both species were initially cultured in the defined, iPSC-specific media mTeSR1 (STEMCELL, Vancouver, Canada). In order to investigate interspecies differences in CT-specific gene expression between humans and chimpanzees, we generated RNA-seq data from human and chimpanzee NPCs, neurons, and astrocytes from iPSCs (Fig. 1a).

To confirm that expression profiles of our iPSC-derived neural samples resembled that of neural tissue and primary neural cells, we used previously published data from human and chimpanzee tissues, including brain (Brawand et al. 2011), as well as human primary neurons and astrocytes (Materials and Methods) (Zhang et al. 2016). We visualized these data in an multidimensional scaling (MDS) plot and observed that our iPSC-derived neural samples clustered together within the same dimensional space as the other neural tissue and cell samples and not the nonneuronal tissue samples (supplementary fig. S5, Supplementary Material online). These clustering analyses demonstrate that we successfully created and obtained total transcriptome data of iPSC-derived NPCs, neurons, and astrocytes from humans and chimpanzees relevant for comparative assessments of CT-specific interspecies gene expression differences.

### DE Between Human and Chimpanzee Neural CTs

We next performed DE analyses in order to determine significantly differentially expressed genes between species. However, given the lack of clear distinction among our different CTs (Fig. 1b), we first wanted to determine the degree of shared expression across all CTs. To do so, we determined overlap among CTs for genes with at least 1 count in 1 or more cell lines per CT (Fig. 1c). Of the total genes expressed in NPC (*n* = 14,877), neuron (*n* = 14,961), and astrocyte (*n* = 14,931) samples, 95.13% (*n* = 14,536) were shared among all 3 CTs (Fig. 1c). This is consistent with previous findings that relatively few genes are CT-specific in the brain, in terms of absolute expression (Magistretti and Allaman 2015; McKenzie et al. 2018).

For DE analyses, we first conducted an analysis of variance (ANOVA)-like test for differentially expressed genes in a species (SP) by CT manner using edgeR (Robinson et al. 2010). We reasoned that this would be the most evolutionarily relevant set of genes for investigating neural CT-specific “trade-offs” in expression between species. Using edgeR's generalized linear model (GLM) functionality and a quasi-likelihood *F*-test for significant DE, we found 4,007 significantly differentially expressed genes in a species by CT manner (26.22% of all expressed genes). However, at present, there are no post hoc tests for an ANOVA-like test for DE, and so this analysis is limited in that it cannot delineate which samples (CTs) these genes are significantly DE in (Robinson et al. 2010). The ANOVA-like test for differences also requires an initial filtering of lowly expressed genes across all samples, which eliminates the 104 to 120 genes (0.68% to 0.79%; Fig. 1c) expressed only in 1 CT (CT-specific genes). For these reasons, we also conducted interspecies pairwise DE comparisons for each CT (hereafter referred to as CT-DE analyses). While these CT-specific genes are relatively few in number, they likely have an important role in cellular function, and thus, we did not want to exclude them from our interspecies CT-DE comparisons.

**Fig. 2.**
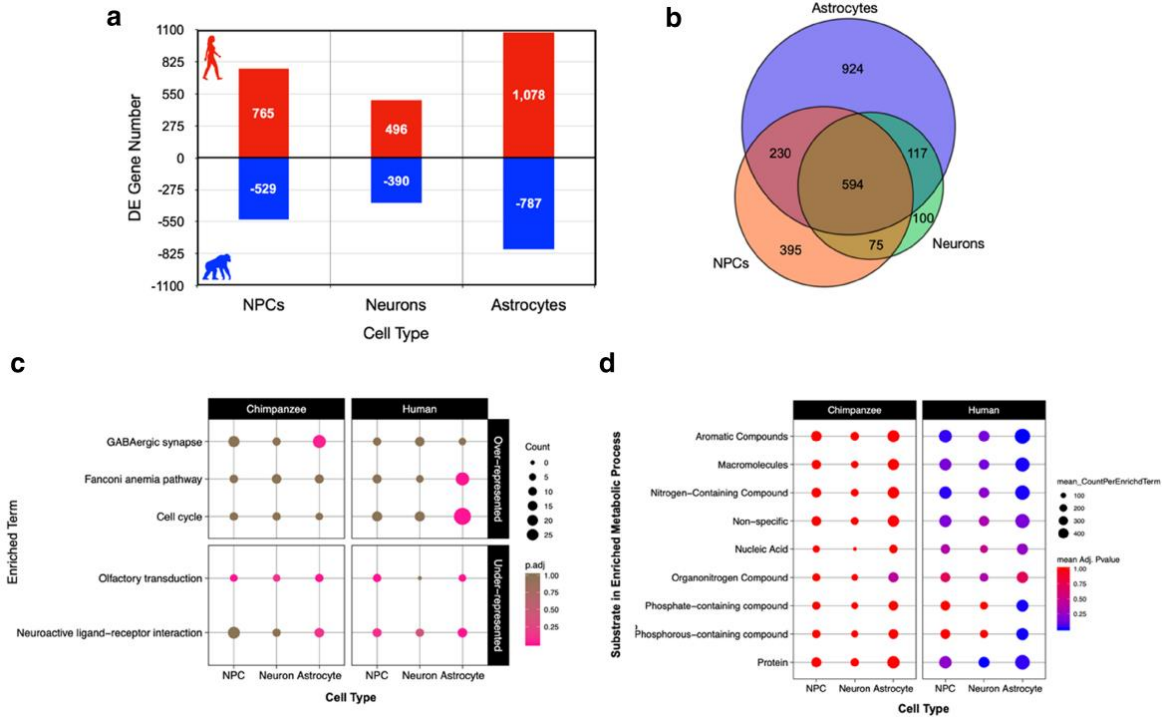
Astrocytes demonstrate the most significant differences in gene expression between human and chimpanzee neural CTs for metabolic but not neuron-specific pathways. a) Counts of genes exhibiting DE at an FDR < 5% between species for each CT and the direction of higher expression for each CT-DE comparison (red/up/positive, higher expression in human; blue/down/negative, higher expression in chimpanzee). b) A Venn diagram of overlap in genes per CT exhibiting DE between species. c) Plot of significantly (q < 0.05) overrepresented (top panel) and underrepresented (bottom panel) KEGG pathways determined by categorical enrichment analyses. Size indicates the count of genes per pathway, while color indicates the adjusted *P*-value (pink, lower/significant; brown, higher/nonsignificant). d) Plot of significantly overrepresented categories of GO BP terms determined by categorical enrichment analyses. The categories (*y* axis) represent groupings of multiple GO BP terms related to the metabolism of the indicated substrates/macromolecules. Size indicates the mean count, and color indicates the mean adjusted enrichment *P*-value for all terms in that category.

For CT-DE comparisons, the only genes included were those counts above zero in all samples per CT and were further filtered to those with counts per million (CPM) > 1 in at least 1 sample, resulting in 11,772 genes in NPCs, 12,451 genes in neurons, and 12,302 genes expressed in astrocytes. We used the same GLM quasi-likelihood *F*-test to determine that 8.57% (*n* = 1,294) of genes are differentially expressed between species’ NPCs, 5.8% (*n* = 886) between neurons, and 12.2% (*n* = 1,865) between astrocytes (Fig. 2a, supplementary fig. S6, Supplementary Material online). Many of these significantly differentially expressed genes in CT-DE comparisons overlapped with the SP × CT ANOVA-like differentially expressed genes (supplementary table S3, Supplementary Material online). When we determined overlap in differentially expressed genes between species across all 3 CTs, we found that, similar to global expression, a large number of genes were determined as differentially expressed between species in all 3 CTs (*n* = 594; Fig. 2b). However, there are far more genes that uniquely differentiate astrocyte gene expression between species (*n* = 924) than NPCs (*n* = 395) and neurons (*n* = 100) (Fig. 2b). This suggests that neuronal gene expression is more conserved across species in NPCs and neurons and that astrocytes do indeed contribute to important interspecies differences in neural gene expression.

### Interspecies Differences in Gene Expression Are Largely Due to Differential Metabolic Signaling Skewed Toward Higher Expression in Humans Regardless of CT

We then used categorical enrichment analyses to determine what biological processes (BPs) are overrepresented (enriched) or underrepresented (“conserved”) in interspecies differentially expressed genes by CT (supplementary table S4, Supplementary Material online). There were consistently a larger number of interspecies differentially expressed genes per CT-DE comparison with higher expression in human cells (765 in NPCs, 496 in neurons, and 1,078 in astrocytes) than chimpanzee cells (529 in NPCs, 390 in neurons, and 787 in astrocytes) (Fig. 2a). We used these 6 higher-in-1-species split DE gene lists in a multiquery categorical enrichment analyses for under- and overrepresented processes using g:Profiler's categorical enrichment tool (GOSt) (Raudvere et al. 2019). Likely in part due to the larger number of genes with higher expression in human for all CT's, there was consistently far more processes enriched in human CTs than chimpanzee CTs (supplementary table S4, Supplementary Material online).

Human and chimpanzee neural cells exhibited significant underrepresentation of the Kyoto Encyclopedia of Genes and Genomes (KEGG) pathways “olfactory transduction” and “neuroactive ligand–receptor interaction” (Fig. 2c). Consistently, both species also showed significant underrepresentation of Gene Ontology (GO) BP related to development, immune function, and intracellular signaling (supplementary fig. S8, Supplementary Material online). Human cells exhibited underrepresented for some extracellular and membrane-associated cellular components (CC) (supplementary fig. S9, Supplementary Material online) as well as molecular function (MF) related to cytokine and receptor activity (primarily in astrocytes; SI 0). Both species cells were underrepresented for nucleic acid binding and G-protein–coupled receptor and transducer activity (supplementary fig. S10, Supplementary Material online). This demonstrates that signaling, including some neuronal-specific signaling such as neuroactive ligand–receptor interaction, and downstream perception processes (e.g. olfaction) are conserved across species for all CTs.

As for significantly overrepresented processes in differentially expressed genes between species, cell division, cytoskeletal, developmental, signaling, and response to external stimuli terms were significantly overrepresented in human astrocytes, transcription was enriched in human NPCs, and protein modification was enriched most significantly in human neurons (supplementary fig. S8, Supplementary Material online). We were particularly interested how pathways involved in cellular respiration and metabolism differed between species. Metabolic processes targeting a variety of substrates or macromolecules were enriched primarily in human cells (summarized in Fig. 2d, full results in supplementary table S4, Supplementary Material online). Human astrocytes were significantly enriched for several more metabolic BPs than human NPCs and neurons that included metabolism of phosphate-containing compounds as well as more generally for metabolism of macromolecules (Fig. 2d). We also investigated enrichment of GO CC terms to determine if there were differences in expression of specific neuronal parts. Overrepresented CC terms in human astrocytes were similar to the GO BP overrepresented processes (cytoplasm, cytoskeleton, cell division, and growth; supplementary fig. S9, Supplementary Material online). Human neurons were enriched for terms related to intracellular macromolecule modification and trafficking (supplementary fig. S9b, Supplementary Material online). Interestingly, human astrocytes were enriched for MFs related generally to substrate binding and specifically to ATP, carbohydrates and their derivatives, enzymes, and nucleic acids (supplementary fig. S10a, Supplementary Material online). Human neurons for ubiquitin-related molecular activity and human astrocytes for molecular activity related generally to ATPases, catalysis, exonucleases, helicases, kinases, and phosphotransferases (supplementary fig. S10b, Supplementary Material online). These results indicate that metabolic processes differ between species in a CT-specific manner and that all human neural CTs exhibit increased expression for a variety of macromolecular metabolic processes more than chimpanzee neural cells. Further, we see that there are significant differences in MFs important in cellular metabolic signaling.

### Human and Chimpanzee Neural Cells Differ in Glucose and Lactate Transport As Well As OXPHOS

**Fig. 3.**
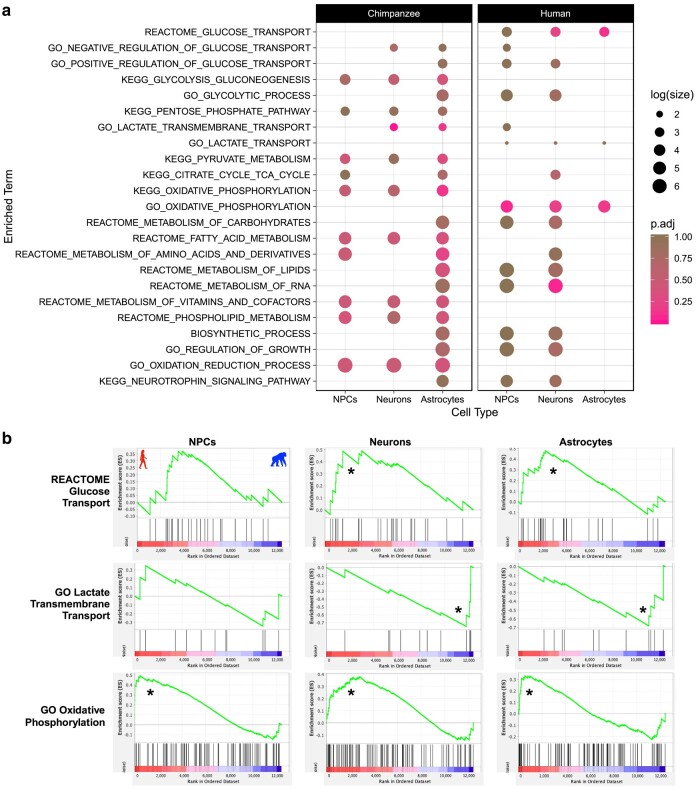
Humans and chimpanzees differ in metabolite transport and OXPHOS in a neural CT manner. a) Plot of all tested gene sets in the GSEA (full results in supplementary table S5, Supplementary Material online). Separate panels indicate which species “phenotype” the gene set was enriched in. Color indicates the FDR Q-value (FDR < 25% indicates significance in this analysis). Size indicates the log(count) of genes included in the enriched gene set. b) Enrichment plots of significant pathways from GSEA for a subset of panel a. The green line indicates the running enrichment score for each gene in the gene set as the analysis moves down the ranked list of genes. The enrichment score for the gene set is the peak of this curve, and an (*) indicates significantly enriched. The bottom panel is the ranked order of the genes and shows their location within that ranked set of genes. The left side of plot (and red/left portion of ranked order plot below) indicates human enrichment, while the opposite (right/blue) indicates chimpanzee enrichment.

Metabolic processes targeting a variety of substrates or macromolecules were enriched in human neural CTs using uninformed categorical enrichment analyses. However, very few of these processes were for pathways involved in cellular respiration resulting in production of energy in the form of ATP. We were interested if there were significant interspecies differences in gene expression for pathways involved in cellular respiration, specifically, those involved in aerobic glycolysis. Because there are known differences in metabolic capacity between neurons and astrocytes, including that astrocytes are characterized metabolically by high aerobic glycolytic activity (increased glycolysis with limited potential for oxidative ATP production), while neurons typically favor energy production and OXPHOS (reviewed in Magistretti and Allaman (2015)), we were interested in determining any interspecies and CT-specific differences in these brain metabolic processes. We should note that we see generally equal amounts of up- and downregulated DE in humans and chimpanzees in the entire data set and that these metabolic pathways were of interest due to the GO enrichments. To further investigate metabolic gene sets, we used a gene set enrichment analysis (GSEA) (Subramanian et al. 2005) with 23 a priori gene sets on the raw counts of the 12,407 genes used for interspecies pairwise CT-DE analyses. Gene sets were obtained from the Molecular Signatures Database (MSigDB) (Liberzon et al. 2011) and chosen in order to probe a variety of energetic metabolic pathways and substrate transporters of varying gene number sizes from multiple ontology categories (GO, KEGG, and REACTOME) (all probed gene sets listed in Fig. 3a) (Ogata et al. 1999; Ashburner et al. 2000; The Gene Ontology Consortium 2017; Fabregat et al. 2018). The goal was to determine if pathways involved in aerobic glycolysis (e.g. OXPHOS, glucose transport, and the citric acid [TCA] cycle) differ in a species by CT manner, and so pathways not directly involved in aerobic glycolysis (e.g. fatty acid metabolism) are included as a comparison. We also included “control” pathways not directly related to metabolism (regulation of growth and neurotrophin signaling).

Our GSEA results indicate that the gene sets for lactate transmembrane transport, glucose transport, OXPHOS, and metabolism of RNA are significantly different between human and chimpanzee neural cells (false discovery rate [FDR] < 25% and nominal *P*-value < 0.05; Fig. 3a and b, supplementary table S5, Supplementary Material online). Glucose transport was enriched in human neurons and astrocytes, while lactate transmembrane transport was enriched in chimpanzee neurons and astrocytes (Fig. 3a and b). The GO gene set for OXPHOS is significantly enriched in all human CTs, while the KEGG OXPHOS is upregulated in chimpanzee astrocytes (Fig. 3a and b). These results indicate these metabolic pathways differ in expression in human and chimpanzee cells in a CT-specific manner. In vitro experiments will help to understand what specific changes are being driven by this divergent gene expression.

### There Are Species by CT Differences in Expression of OXPHOS Protein Complexes

**Fig. 4.**
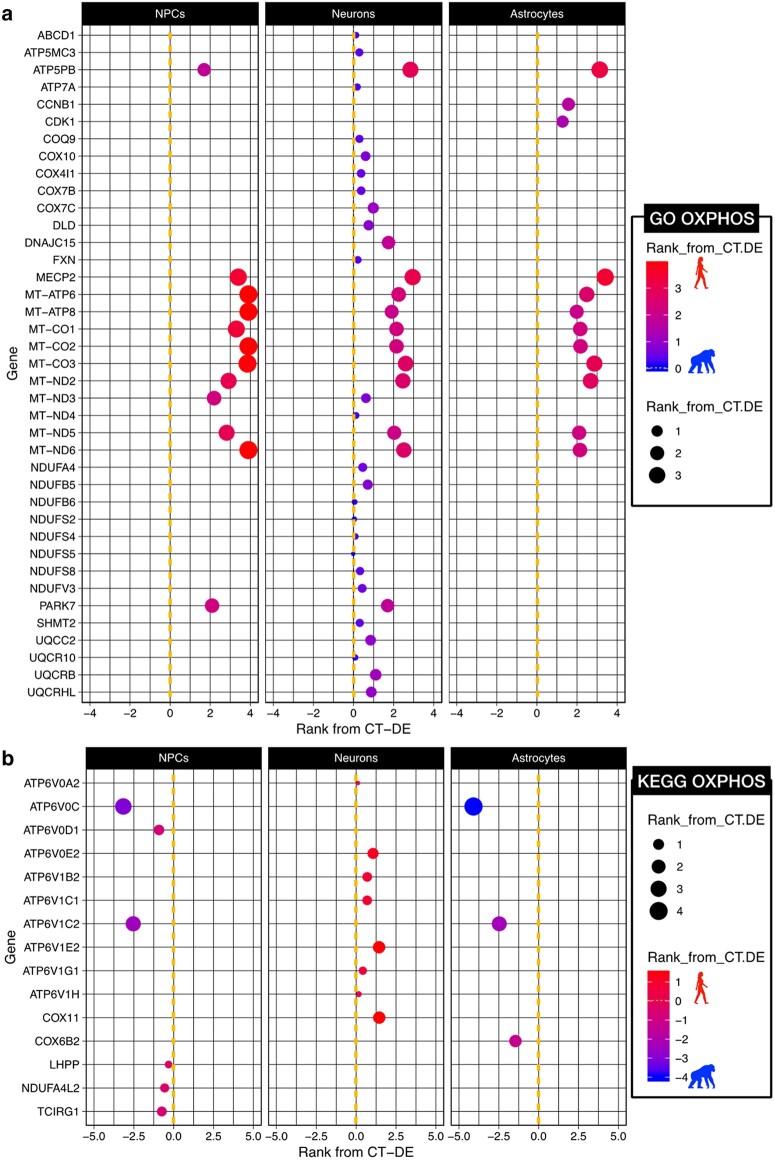
Interspecies expression differences of OXPHOS genes is influenced by higher expression of mitochondrial genes in all neural human CTs. CT-DE results of genes per a) GO or b) KEGG OXPHOS gene sets determined as members of the core set of genes influencing significant enrichment of these gene sets in the GSEA analysis. CT-DE rank was calculated per each gene [(sign of logFC) × log10(FDR Q-value)], with values >0 indicating higher expression in human and values <0 indicating higher expression in chimpanzee. Color spectrum and size also indicate rank (red, higher in human; blue, higher in chimpanzee; larger = higher rank).

**Fig. 5.**
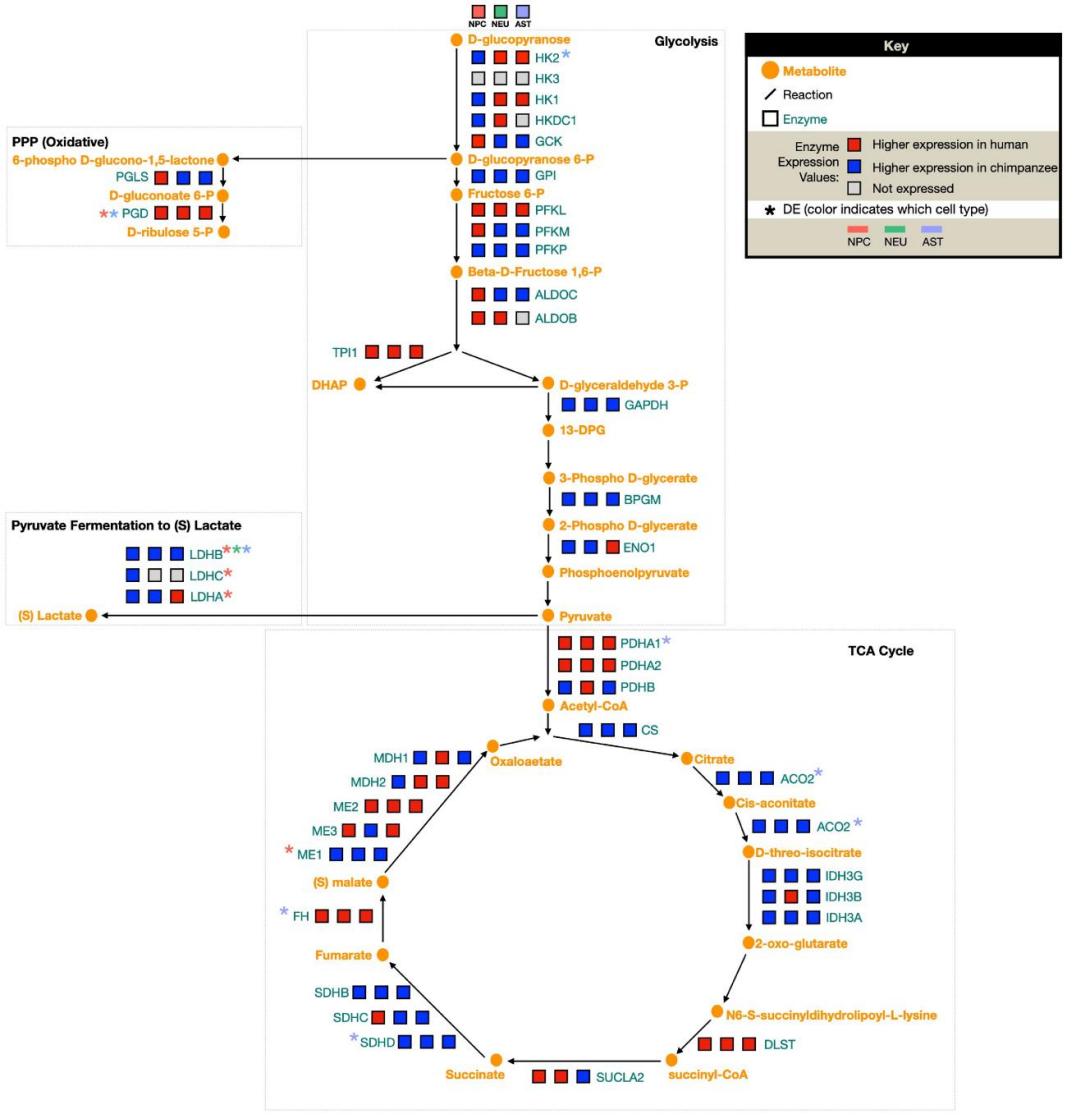
Divergence in pyruvate utilization between species’ astrocytes. We constructed a focal set of aerobic glycolysis signaling pathways in order to contextualize our DE results in the framework of a network signaling. A diagram of the major pathways involved in aerobic glycolysis (glycolysis, PPP, lactate conversion from pyruvate, and TCA cycle). For each enzyme in the pathway, 3 blocks indicate expression of this enzyme in each CT—left to right: NPCs, neurons, and astrocytes. Color indicates the level of expression (higher in human [red], higher in chimpanzee [blue], not expressed in this CT [grey]).

Leading edge analyses of significant GSEA gene sets are used to determine which genes of the gene set contribute most strongly to the enrichment of that pathway in the phenotype (Subramanian et al. 2005). We examined the results from the leading edge GSEA analysis with CT-DE expression analyses to get a better idea of how these 3 pathways diverge in a CT by species manner (Figs. 4 and 5; full results in supplementary table S6, Supplementary Material online). We calculated a rank for DE genes for each CT-DE comparison (NPC, neuron, and astrocyte): (sign of logFC) × log10(FDR Q-value) (Reimand et al. 2019) and used that in addition to the GSEA leading edge analysis to determine significant differences. For the OXPHOS genes, we were interested in determining why there was a difference in species and CT enrichment based on the source of the gene set (KEGG vs. GO) and determining if there were potential functional differences in OXPHOS between species. We mapped the CT-DE rank of the core-enriched genes for GO (Fig. 4a) and KEGG (Fig. 4b) OXPHOS genes.

The core set of genes in GO and KEGG OXPHOS gene sets included genes for subunits of cytochrome c oxidase (the nuclear-encoded *COX4I1*, *COX6B2*, *COX7B*, and *COX7C* and the mitochondrially encoded *MT-CO1*, *MT-CO2*, and *MT-CO3*) as well as those that aid in cytochrome c oxidase assembly (*COX10* and *COX11*) (Fig. 4a and b; supplementary table S6, Supplementary Material online). Cytochrome C oxidase is the terminal complex in the electron transport chain and is crucial to maintaining a proton gradient across the inner mitochondrial membrane for ATPase to synthesize ATP. These genes are of particular interest, because within anthropoids, genes encoding the subunits of cytochrome c oxidase show an accelerated rate of evolution in their sequences compared with any other placental mammals (Preuss 2012). Here, we see a CT by species divergence in cytochrome c oxidase gene expression, where most of these genes exhibit higher expression in human neurons (Fig. 4a). There is also a clear trend of mitochondrially encoded genes that function in OXPHOS having significantly higher expression in human cells, including those for cytochrome c oxidase subunits (*MT-CO1*, *MT-CO2*, *MT-CO3*) but also mitochondrially encoded ATP synthase (*MT-ATP6*) and mitochondrially encoded subunits of the NADH:ubiquinone oxidoreductase core of electron transport chain complex I (*MT-ND2*, *MT-ND3*, *MT-ND5*, and *MT-ND6*) (Fig. 4a).

A major difference between the GO and KEGG OXPHOS gene sets is that the KEGG OXPHOS set includes vacuolar-ATPase (V-ATPase) genes, whose major role is in acidification of intracellular organelles and have an important function in synaptic vesicle proton gradient formation and maintenance (Maxson and Grinstein 2014; Pamarthy et al. 2018). There is an intriguing pattern of enrichment for higher expression of subunits of V-ATPases in a CT by species manner (Fig. 4). Three genes for subunits of V-ATPases (*ATP6V0C*, *ATP6V0D1*, and *ATP6V1C2*) are core-enriched genes in the KEGG OXPHOS gene set and are significantly enriched in CT-DE with higher expression in chimpanzee NPCs and astrocytes but not neurons (Fig. 4b). Furthermore, only one of these V-ATPase genes is DE in a SP × CT manner (*ATP6V1C2*) (Fig. 4b). However, several other V-ATPase subunit genes are core-enriched only in human neurons (Fig. 4b), most notably *ATPV1E2* and *ATPV0E2*, both of which are core-enriched and significantly differentially expressed in human neurons. This shows that V-ATPases exhibit significant DE between humans and chimpanzees and that human neurons are distinct in V-ATPase gene expression from chimpanzee NPCs and astrocytes.

### Interspecies DE of Important Metabolite Transporter Genes in Neurons and Astrocytes

It is widely accepted that neurons exhibit limited glycolytic capacity and that astrocytes respond to signals associated with increased synaptic signaling by increasing glucose uptake and subsequent aerobic glycolysis of glucose to produce lactate to be used as energy source by neurons (Pellerin and Magistretti 1994; reviewed in Magistretti and Allaman (2015)). For this reason, we were interested in investigating if there were interspecies gene expression differences in lactate transport, particularly in neurons and astrocytes. The GO gene set “lactate transmembrane transport” was enriched in chimpanzee neurons and astrocytes, showing that genes involved in lactate transport are more highly expressed in these mature CTs than NPCs (supplementary fig. S11a, Supplementary Material online). Several genes in this gene set are for proton-linked monocarboxylate transporters that transport pyruvate and lactate (*SLC16A11*, *SLC16A12*, *SLC16A13*, and *SLC16A6*) that are all core-enriched in neurons and astrocytes (supplementary fig. S11a, Supplementary Material online). *SLC6A11* and *SLC16A13* are also differentially expressed in SP × CT ANOVA-like DE as well as a CT-DE manner, though *SLC16A13* is not in astrocytes (supplementary fig. S11a, Supplementary Material online). The enrichment for the lactate transmembrane transport gene set in chimpanzee neurons and astrocytes and the corresponding DE of specific pyruvate and transporter genes between species might suggest that chimpanzee neurons and astrocytes have the capacity to shuttle pyruvate and lactate at a higher rate than human neurons and astrocytes.

In addition to lactate transmembrane transport enrichments, the glucose transport gene set was significantly enriched in human neurons and astrocytes (Fig. 3). There were 2 hexokinase genes (*HK1* and *HK2*) core-enriched in this gene set that demonstrate lower expression in chimpanzee NPCs but higher expression in human astrocytes (supplementary fig. S11b, Supplementary Material online), though only *HK2* is significantly upregulated in human astrocytes by CT-DE analysis (supplementary fig. S11b, Supplementary Material online). *G6PC3* may not be significantly differentially expressed in any particular CT, but it is in the SP × CT-DE comparison and does show insignificant but consistently higher expression in all chimpanzee CTs (supplementary fig. S11b, Supplementary Material online). *SLC2A3* is a facilitative glucose transporter across the cell membrane, and here, it exhibits core enrichment in all 3 human CTs by the GSEA leading edge analysis, as well as moderately (though nonsignificant) higher expression in human (supplementary fig. S11b, Supplementary Material online). The enrichments of glucose transport in human neurons and astrocytes appears to be influenced by increased expression of plasma membrane–associated glucose transporters (e.g. *SLC2A3*) and enzymes that function in the earlier steps of glycolysis (*HK1*, *HK2*, *G6PC3*).

### Intersecting Genes With DE and Putative Signals of Positive Selection

In order to begin to probe whether expression differences between species are influenced by selective pressures, we obtained synonymous (dS) and nonsynonymous (dN) nucleotide mutation rates from Ensembl (Kersey et al. 2017; Schneider et al. 2017) and compared the rate of change (dN/dS) for different groups of iPSC-derived neural cell expressed genes. A dN/dS > 1 indicates putative evidence of positive selection in coding regions (Herrero et al. 2016). As predicted, the vast majority of all the genes identified as expressed in these cells did not exhibit a dN/dS > 1 (supplementary fig. S12, Supplementary Material online). Only few genes DE between species in iPSC-derived astrocytes (*n* = 6), neurons (*n* = 6), and NPCs (*n* = 11) exhibit signs of coding selection (dN/dS > 1) (supplementary table S7 and fig. S12, Supplementary Material online). The gene histidine-rich calcium-binding protein (*HRC*) exhibits positive selection and is significantly DE between species in NPCs, but not astrocytes, and is not expressed at all in neurons (supplementary table S7, Supplementary Material online). Three genes (*DCTN6*, *HHLA3*, *DBNDD2*) have a dN/dS > 1and showed significantly DE in all 3 CTs (supplementary table S7, Supplementary Material online). However, there is no commonality in these genes to suggest any meaningful impact on gene expression differences or in specific CTs.

In a separate analysis, we chose to look at a much smaller subset of genes to test for signals of positive selection in putative *cis*-regulatory regions upstream of the coding sequence (see Materials and Methods). We tested the promoter regions of 156 aerobic glycolysis genes that were expressed in our samples. Thirteen of 156 aerobic glycolysis genes exhibited signs of positive selection (supplementary table S8, Supplementary Material online). These included 2 V-ATPase component proteins (*ATP6V1G1* and *ATP6V1H*), 4 nucleoporins (*NUP85*, *NUP54*, *NUP214*, and *NUP107*), a subunit of the NADH dehydrogenase complex of the ETC (*NDUFA4*), cyclin B1 (*CCNB1*), an RNA binding protein (*RAE1*), and 2 glycolysis genes, glucokinase regulator (*GCKR*) and hexokinase (*HK1*). Interestingly, though all of these genes were expressed to some degree in all 3 CTs and in both species (with the exception of *GCKR*), they were only ever significantly differentially expressed between species in astrocytes (*n* = 4 DE in astrocytes; supplementary table S8, Supplementary Material online). Of these 4 genes that were significantly DE between species and under positive selection, *CCNB1*, *NDUFA4*, and *NUP85* were more highly expressed in human astrocytes, while *SLC16A11* was more highly expressed in chimpanzee astrocytes. Of note, *GCKR* is only expressed in astrocytes, with significantly higher expression in chimpanzee (supplementary table S8, Supplementary Material online). Significant results from this test suggest regulatory elements that control expression of these genes may be under selection in humans. Significant results from this analysis support selection in genes involved in metabolic processes in humans.

### Differences in the Aerobic Glycolysis Genes Are Primarily in NPCs and Astrocytes But Not Neurons

In order to obtain a pathway-level understanding of altered expression of aerobic glycolysis in iPSC-derived neural cells between humans and chimpanzees, we reconstructed a signaling network diagram of enzymes involved in 4 subpathways involved in aerobic glycolysis (glycolysis, PPP, pyruvate conversion to lactate, and TCA cycle) from the HumanCyC database (Romero et al. 2005) (Fig. 5). We then mapped discrete expression values (higher in human, higher in chimpanzee, not expressed) for each of these enzymes in all 3 CTs onto the pathway diagram to illustrate which species the enzymes were more highly expressed in and if they were significantly differentially expressed between species. From this, we see dynamic changes in expression across aerobic glycolysis subpathways, with no significant shift toward higher expression of enzymes in 1 species or CT at any of these subpathways (Fig. 5). Aerobic glycolysis enzymes exhibiting interspecies DE in NPCs were *PGD*, *LDHA*, *LDHB*, *LDHC*, and *ME1*; enzymes demonstrating DE in astrocytes were *PGD*, *HK2*, *PDHA1*, *FH*, *ACO2*, and *SDHD*; and only a single enzyme exhibited interspecies DE in neurons (*LDHB*) (Fig. 5). This shows that NPCs and astrocytes, but not neurons, exhibit the vast majority of significant differences in expression of enzymes in these pathways (Fig. 5). The majority of these genes was expressed in all CTs, particularly those that exhibited significant DE in NPCs or astrocytes, so this lack of DE in neurons is not simply due to CT-specific expression differences (supplementary fig. S13, Supplementary Material online). Human and chimpanzee astrocytes appear to diverge at the stage of pyruvate utilization, where human astrocytes exhibit significantly higher expression of *PDHA1*, which converts pyruvate into acetyl-coA, whereas chimpanzee astrocytes show significantly higher expression of *LDHB*, which converts pyruvate into lactate rather than acetyl-coA (Fig. 5). Interestingly, *LDHB* is also the only enzyme in these pathways exhibiting DE between species in neurons. Other *LDH* isoforms (*LDHC* and *LDHA*) also exhibit significant interspecies DE, with higher expression in chimpanzee NPCs. All chimpanzee neural CTs differ from human chimpanzees for *LDH* expression, but chimpanzee NPCs differ from human NPCs in expression levels of multiple *LDH* isoforms. This pathway-level consideration of expression differences between species suggests significant changes in aerobic glycolysis enzyme activity primarily in NPCs and astrocytes and an interspecies divergence in pyruvate utilization.

## Discussion

Our novel approach using iPSCs allowed us to investigate rare neural CTs from primates to determine CT-specific changes in gene expression in genes involved in metabolic pathways which may have been necessary to support evolution of the human brain. Our results demonstrate that interspecies divergence in gene expression is more conserved in neurons and significantly greater between species’ astrocytes. DE between species’ CTs is enriched for metabolic processes related to cellular respiration. This finding is similar to that of previous studies of DE between human and chimpanzee whole brain tissue (Haygood et al. 2007; Kosiol et al. 2008; Uddin et al. 2008) and is driven primarily by higher expression of metabolic genes in human cells. However, there were some interesting examples of potential trade-offs in expression patterns of specific genes and pathways in a CT by species manner. We determined that human neurons and astrocytes are enriched for higher expression of glucose transport proteins while chimpanzee neurons and astrocytes exhibit higher expression of lactate transmembrane transport genes and that there are dynamic interspecies changes in expression of nuclear- and mitochondrially encoded subunits of the protein complexes important for OXPHOS. Our study demonstrates the utility of iPSC-derived cells for better understanding evolution of gene expression in primate brains.

Previous work has determined several significant differences in expression of cellular respiration pathways and evidence of differential selective pressure associated with metabolic genes (both noncoding and coding) between human and chimpanzee brains (Wu et al. 1997; Grossman et al. 2001; Wildman et al. 2002; Goldberg et al. 2003; Grossman et al. 2004; Uddin et al. 2008; Hüttemann et al. 2012). However, the heterogenous nature of brain tissue has complicated drawing specific conclusions about role of specific CTs on this trajectory of elevated metabolic expression in human brains. Specifically, there is a long-standing question about the sole influence of greater neuron numbers in human brains (Herculano-Houzel 2011) on the observed increase in glucose utilization (Mink et al. 1981; Hofman 1983). Using iPSC-derived neural CTs and comparing gene expression differences in a CT by species manner allowed us to test interspecies differences in the CT-specific contribution of metabolism to long-understood differences in brain metabolic capacity. Because our methods of determining significant differences in gene expression do not rely on number of cells or quantity of transcripts, we are able to conclude that there are CT-specific contributions to altered metabolic gene expression between species’ neural cell types and that the sheer number of cells alone likely does not fully explain metabolic differences between human and chimpanzee brains. Furthermore, our results demonstrate that, in light of the relatively recent discovery of CT-specific metabolic differences between neurons and astrocytes, investigation of differences in brain metabolism among primates and the evolutionary processes that shaped them would indeed be incomplete without the consideration of all metabolically relevant neural CTs, not just neurons. We found that astrocytes demonstrate the greatest proportion of interspecies difference in metabolic gene expression and that neuronal gene expression appears to be more conserved across species. This suggests that astrocyte-mediated differences in metabolic brain function may be an important mechanism by which the ultimate evolutionary trajectory of human brain evolution has occurred.

Human cells show increased capacity for glucose transport, via greater expression of glucose transporters, in the mature neural CTs. This may suggest that the observed differences in glucose utilization by the human brain extend beyond development and may play an important role in more mature neurons and astrocytes for either energy or macromolecule production. Furthermore, lactose dehydrogenase (*LDH*) isoforms favor differential affinities for interconverting pyruvate and lactate. *LDHB* favors the production of lactate into pyruvate (Bauernfeind and Babbitt 2014; Almad et al. 2016) and is significantly differentially expressed with higher expression in chimpanzee for all CTs. This coupled with higher expression of lactate transporters in chimpanzee astrocytes suggests that chimpanzee cells may be favoring production and transport of lactate at a higher rate than all human neural CTs tested. This opposing enrichment for elevated glucose transport in mature human neural CTs in comparison with elevated lactate transport and conversion to pyruvate in mature chimpanzee cells raises some intriguing questions about metabolic trade-offs between human and chimpanzee brains. If we presume that the direction of change in metabolic gene expression is on the human lineage and we do have some evidence from signs of positive selection on glucose and energetic metabolism coding and noncoding genes within primates with proximity to humans (Grossman et al. 2001; Goldberg et al. 2003; Grossman et al. 2004; Uddin et al. 2008; Hüttemann et al. 2012), then perhaps an increase in glucose uptake in human brains has allowed for a decrease in expression of genes that convert and shuttle lactate (via LDH and lactate transporters) to produce pyruvate. Previous studies have found lineage-dependent differences in enrichments for metabolic pathways in genes DE between primate brain regions, with greater glucose and carbohydrate metabolism in humans but higher glycogen and acyl-CoA metabolism in chimpanzees (Haygood et al. 2007; Kosiol et al. 2008; Uddin et al. 2008). The increase in LDH and lactate transport in chimpanzee neurons and astrocytes may be the CT-specific mechanism responsible for findings of significant metabolic differences in previous studies of whole brain tissue.

Human neural cells were enriched at the pathway level for OXPHOS genes, and within that pathway, there were some interesting examples of opposing enrichment for subunits of OXPHOS protein complexes. We observed increased expression and enrichment for components of cytochrome c oxidase, which previous studies have determined genes involved in this complex to be under positive selection (Goldberg et al. 2003). However, we expand on the knowledge of interspecies differences in cellular respiration complex expression by determining that these components are higher in human in all CTs investigated and particularly in human neurons (Fig. 4a). We also observed higher expression for subunits of other electron transport chain complexes, including ATP synthase and the NADH:ubiquinone oxidoreductase components of complex I. This CT by species approach also allowed for us to determine that human neurons and chimpanzee NPCs and astrocytes have higher expression for genes involved in V-ATPase function. Astrocytes respond to signals associated with increased synaptic signaling (Pellerin and Magistretti 1994; reviewed in Magistretti and Allaman (2015)) by increasing glucose uptake and subsequent aerobic glycolysis of that glucose to lactate to be used as an energy source for neurons. Our findings that V-ATPases are significantly differentially expressed between humans and chimpanzees suggest that human neurons are distinct in V-ATPase gene expression from chimpanzee NPCs and astrocytes. Given the important function of V-ATPases in synaptic vesicle formation for neurotransmitter signaling, this may be a mechanism by which human-specific changes in neuronal signaling have occurred.

Our investigation into the overlap of signatures of positive selection in coding regions of genes exhibiting interspecies DE revealed very little new or intriguing information. The dN and dS scores obtained from Ensembl for use in this analysis were averages across all sites in a given gene, thus minimizing significant changes at specific sites (Yang 2007). Importantly, it necessary to investigate positive selection in both coding and noncoding regulatory regions (i.e. promoters and *cis*-regulatory regions) associated with metabolic genes, as changes in both gene sequence and gene expression can confer adaptive phenotypic differences (Haygood et al. 2007; Kosiol et al. 2008; Uddin et al. 2008; Haygood et al. 2010; Bauernfeind et al. 2015). We chose to look at a much smaller subset of genes to test for signals of positive selection in putative *cis*-regulatory regions upstream than the genome-wide values for coding selection. Noncoding regulatory regions of genes in OXPHOS and glycolysis pathways are found to be under positive selection to varying degrees in humans (Haygood et al. 2010). A specific example is glucose-6-phosphate isomerase (*GPI*), which has been determined to be under positive selection in its noncoding promoter region in previous studies (Haygood et al. 2007); however, it does not exhibit interspecies DE in any of these CTs. However, we did find evidence of positive selection in promoters of several aerobic glycolysis genes in the human lineage. Interestingly, we see that aerobic glycolysis genes exhibiting positive selection in promoter sequences were only significantly differentially expressed between species in astrocytes, not NPCs or neurons. Hexokinase and glucokinase both function in the conversion of glucose to glucose-6-phosphate, thus playing an early role in glycolysis. We found that there are significant expression differences in these key genes between species and only in astrocytes. Human astrocytes significantly upregulate *HK1*, while chimpanzee astrocytes significantly upregulate *GCKR*, which also exhibits positive selection in its promoter. This suggests that there is an evolved difference in the initial processing of glucose during glycolysis in an astrocyte-mediated manner, in addition to interspecies differences in the expression of glucose transporters. There is also evidence of adaptive divergent astrocyte glycolytic activity between species in pyruvate utilization. In addition to the difference in pyruvate conversion enzymes and lactate transmembrane shuttling, we also found evidence of positive selection on the human lineage and a significant increase in expression in chimpanzee astrocytes of *SLC16A11*, which functions in catalyzing transport of pyruvate across the plasma membrane. These results are intriguing in that they demonstrate evidence of positive selection in the human lineage and divergent gene expression in genes involved in pyruvate processing and transport. These positive selection analyses further corroborate that there are significant differences in glycolytic gene expression between species’ astrocytes at initial steps in glycolysis as well as pyruvate utilization. Combined, this supports the evolution of metabolism in the human brain. Future investigations of the overlap between genes exhibiting DE in a CT-specific manner and signatures of positive selection should utilize methods that allow for branch and site models that are more effective at determining positive selection in a lineage-specific manner (e.g. HyPhy for noncoding and coding regions) (Pond et al. 2005; Haygood et al. 2007; Horvath et al. 2014; Muntané et al. 2015).

Our focal analysis of aerobic glycolysis enzyme expression yielded several important findings. We show that there is not a consistent single species skew in expression levels for any of the subpathways in aerobic glycolysis. The lack of significant DE between species in neurons for aerobic glycolysis enzymes demonstrates the importance of studying CTs other than neurons when investigating human brain evolution and suggests that astrocytes may indeed be critical for the evolution of the metabolically demanding human brain. We also found that it is not simply the lack of glycolytic capacity of neurons (Almeida et al. 2004; Herrero-Mendez et al. 2009; Sonntag et al. 2017) that contributes to CT-specific signaling disparities, at least in a comparative manner. Perhaps the most intriguing finding is the interspecies divergence in processing pyruvate. Humans exhibit significantly higher expression of *PDHA1* than chimpanzees do, indicative of a functionally relevant increase in conversion of pyruvate into acetyl-CoA and further utilization of the products of glycolysis for energy production, while chimpanzee astrocytes exhibit expression phenotypes suggestive of greater lactate production (higher expression of *LDH*, which converts pyruvate to lactate) as well as enrichment for greater lactate transmembrane transport in chimpanzee neural CTs (higher expression of lactate transmembrane transporters). This suggests that chimpanzee neural cells, and most prominently astrocytes, have a significantly greater capacity to convert pyruvate into lactate and then shuttle it across membranes than human astrocytes do. These analyses suggest significant interspecies changes in aerobic glycolysis enzyme activity primarily in NPCs and astrocytes and an interspecies divergence in pyruvate utilization. Previous work has shown a shift from aerobic glycolysis in NPCs to OXPHOS in more mature neurons (Zheng et al. 2016), but this study is the first of our knowledge to compare across species and include astrocytes. More generally, we see that astrocytes exhibit the greatest degree of expression difference between species than the other CTs, while neuronal gene expression is more conserved. A recent investigation of multiple brain regions from human, chimpanzee, bonobo, and macaque using single-cell RNA-seq also found that astrocytes were one of the CTs exhibiting the greatest expression differences in humans (Khrameeva et al. 2020). This increased variation in interspecies gene expression in astrocytes suggests that previously observed differences in whole brain gene expression may be due astrocyte-specific changes to a larger degree than previously thought and that this is a crucial CT to consider when investigating human-specific brain gene expression has evolved.

Other glial CTs are likely also important players in modulation metabolic changes and neuronal activity. Oligodendrocytes have undergone an increased acceleration in the human lineage compared with neurons (Berto et al. 2019), with human-specific oligodendrocyte genes enriched for functional categories such as RNA metabolism and RNA processing. Another study found that both astrocytes and oligodendrocyte progenitors displayed more differences in the human evolutionary lineage than neurons (Khrameeva et al. 2020). It will be important to see how these glial cells interact with neurons in coculturing or other cell-based experiments.

We determined CT by species differences in gene expression for nuclear- and mitochondrially encoded subunits of the protein complexes important for OXPHOS. We demonstrated a significant interspecies divergence in aerobic glycolytic gene expression in astrocytes, suggesting that this traditionally understudied glial CT likely contributes to the tissue-level shifts in gene expression and suggests that astrocytes play an important role in the evolution of the metabolically expensive human brain. A potential challenge in CT-specific studies of interspecies differences in brain gene expression is the loss of intercellular signaling between different CTs, a hallmark of synaptic signaling in whole tissue. Furthermore, the astrocyte–neuron lactate shuttle links the complementary metabolic needs of astrocytes and neurons (Pellerin and Magistretti 1994; reviewed in Magistretti and Allaman (2015)). Neurons are considered largely to lack the ability to increase glycolytic activity (Almeida et al. 2004; Herrero-Mendez et al. 2009; Sonntag et al. 2017). Future studies of gene expression differences with controlled levels of intercellular signaling by building in complexity (e.g. interspecies differences in expression of single CTs compared with that of cocultured iPSC-derived neurons and astrocytes) could further inform interspecies differences in neuronal gene expression.

Evolved differences in metabolic investment may be the basis for a number of primate-specific phenotypes, including those that are unique to humans, such as slow reproduction and growth and correspondingly longer lifespan than other placental mammals (Charnov and Berrigan 1993; Snodgrass et al. 2007; Pontzer et al. 2014). Adaptation can act on metabolic phenotypes through alterations to energy budget including reductions of or increases in total energy budget or differential allocation of energy within energy budget (Speakman 2005; Lovegrove 2009; Pontzer et al. 2014). Our results demonstrate that altered gene expression between species’ astrocytes, an understudied, but critical, brain CT with known metabolic relevance, provides insight into the metabolic changes that were necessary to support evolution of the human brain.

## Materials and Methods

### Samples and Cell Culture

iPSCs from 3 individuals (cell lines) per species (human and chimpanzee) were cultured in defined, iPSC-specific media mTeSR1 (STEMCELL, Vancouver, Canada). These cell lines were originally obtained as fibroblasts from minimally invasive skin biopsies, reprogrammed into iPSCs, and have been extensively validated for their pluripotency and differentiation abilities (Romero et al. 2015; Burrows et al. 2016; Blake et al. 2018; Pavlovic et al. 2018; Ward et al. 2018; Eres et al. 2019; Ward and Gilad 2019). Three cell lines per species, representing 3 male individuals, were used (supplementary table S1, Supplementary Material online). To investigate differences between human and chimpanzee neural CTs, we induced iPSCs from each species first into multipotent, neural-lineage committed NPCs using STEMdiff Neural Induction Medium in monolayer for 3 passages (21 to 28 d), as per manufacturer's instructions (STEMCELL Technologies, Vancouver, Canada). Successful transition of iPSCs into NPCs was determined using immunofluorescence for the absence of the stem cell marker OCT4 and presence of the NPC marker PAX6 (Fig. 1a). NPCs were then expanded into 3 subsets: 1 for RNA collection and 2 for further differentiation and maturation into neurons and astrocytes. We then differentiated NPCs into mature neurons and astrocytes using the neuron- and astrocyte-specific STEMdiff differentiation and maturation kits as recommend by the manufacturer. Briefly, we differentiated NPCs using the STEMdiff Neuron Differentiation Medium for 1 wk and then matured them using the STEMdiff Neuron Maturation Medium for 2 wks. Similarly, we differentiated NPCs using the STEMdiff Astrocyte Differentiation Medium for 3 wks and then matured them using the STEMdiff Astrocyte Maturation Medium for 2 wks. All cells were validated for CT via immunofluorescence prior to harvesting as follows: NPCs for PAX6+/OCT4− (Developmental Studies Hybridoma Bank, University of Iowa), neurons for neuron-specific class III β-tubulin (TUJ1; Neuromics), and astrocytes for glial fibrillary acidic protein (GFAP) (Sigma Aldrich), according to manufacturer's suggestions (supplementary fig. S1, Supplementary Material online). All mature iPSC-derived cells for each CT were harvested at similar timepoints: NPCs at passage 5 to 6 postinduction from iPSCs, mature neurons at passage 3 to 4, and mature astrocytes at passage 5 to 6 postdifferentiation from NPCs and subsequent maturation (supplementary table S1, Supplementary Material online).

We used edgeR (Robinson et al. 2010) to normalize our raw counts across all samples and visualized these data using a MDS plot of all of the expressed genes (Fig. 1b).

### Library Preparation and Sequencing

Total RNA was extracted from cells (1 to 2 wells, 6-well plate) using a RNeasy Plus Mini Kit (Qiagen), including a DNase step to remove residual DNA. Total RNA was analyzed for quality using the Agilent Bioanalyzer system (Agilent RNA 6000 Nano Kit) with RNA integrity numbers (RINs) for all samples between 8.3 and 10 (supplementary table S1, Supplementary Material online). Using the NEBNext Poly(A) Magnetic mRNA Isolation Kit (NEB), mRNA was isolated from intact total RNA, and cDNA libraries were made from each sample using the NEBNext RNA Ultra II Library Prep Kit for Illumina (New England Biolabs). Barcoded samples were sequenced using the Illumina NextSeq 500 platform at the Genomics Resource Core Facility (Institute for Applied Life Sciences, UMass Amherst) to produce 75 base pair single-end reads, yielding a minimum of 32 million reads per sample.

### Read Mapping and Quantification

Quality-filtered reads were aligned to respective species’ most recent Ensembl genome (*Homo sapiens* GRCh38 and *Pan troglodytes* PanTro3.0 (Kersey et al. 2017; Schneider et al. 2017)) with Bowtie2 (Langmead and Salzberg 2012) using default “--local” parameters for gapped alignments, with a minimum alignment percentage of ≥98.84% (supplementary table S1, Supplementary Material online). HT-Seq (Anders et al. 2015) was used to quantify counts per gene for each sample, using Ensembl gene transfer files (GTFs) corresponding to the same genome build used for alignment (Aken et al. 2017). High-quality, 1-to-1 orthologs from *P. troglodytes* were matched to the Ensembl human reference set of genes using BioMart (Kinsella et al. 2011), yielding 15,284 genes identified as expressed in at least 1 sample.

### Clustering Analyses

We used clustering analyses to determine the variation among our iPSC-derived samples as well as in comparison with previously published, publicly available data from other tissues and CTs. For our iPSC-derived samples, we used the R package edgeR (Robinson et al. 2010) to filter out lowly expressed genes (CPM > 1 in 12/17 samples), resulting in 10,715 orthologous genes, and produced an MDS plot of our samples (Fig. 1b). The greatest influence on our samples is species along PC1 and PC2, followed by separation of immature NPC cells from mature CTs (neurons and astrocytes) along PC2 (Fig. 1b). Notably, human samples were more variable than chimpanzee samples. One human cell line (H20961) showed significant variation across all CTs (supplementary figs. S2 to S4, Supplementary Material online); however, the H20961 NPC sample was consistently an outlier, grouping outside of NPCs of either species, and was removed from subsequent analyses. There are no overt technical differences influencing this outgrouping (e.g. individual sex or age, RNA or cDNA library quality, read number, and alignment percentages; supplementary table S1, Supplementary Material online). This cell line has successfully been used in before in other differentiation studies with no overtly different characteristics (Romero et al. 2015; Burrows et al. 2016; Blake et al. 2018; Pavlovic et al. 2018; Ward et al. 2018; Eres et al. 2019; Ward and Gilad 2019).

To compare our samples to previously published data from cells and tissues, we downloaded raw RNA-seq reads from the National Center for Biotechnology Information (NCBI)'s Gene Expression Omnibus (GEO) (Edgar et al. 2002) and processed them from raw read counts through HT-Seq and orthologous gene matching in the same manner as our iPSC-derived samples. To compare our samples with those from primary neural CTs, we used RNA-seq data from primary neurons and astrocytes obtained from 4 hippocampal astrocytes, 4 cortex astrocytes, and 1 cortical neuron from Zhang et al. (2016) (GEO accession number GSE73721) and 3 pyramidal neuron samples (GEO accession numbers GSM2071331, GSM2071332, and GSM2071418) isolated from an unspecified brain region by the ENCODE project (ENCODE Project Consortium 2012; Davis et al. 2018). We also downloaded the tissue-level data from Brawand et al. (2011) (Brawand et al. 2011) from human and chimpanzee brain regions and nonneuronal tissue (heart, kidney, liver) (GEO accession number GSE30352) (supplementary table S2, Supplementary Material online for details). Only genes with counts >0 in all samples were included (*n* = 7,660) and were further filtered to include only those with CPM > 1 in all 23 samples (*n* = 6,124). An MDS plot of normalized counts was generated using edgeR of the top 500 most differentially expressed genes in all samples (supplementary fig. S5, Supplementary Material online).

### Differential Gene Expression Analyses

In order to determine what genes were significantly differentially expressed in a species by CT manner using, we used the R package edgeR's (Robinson et al. 2010) generalize linear model (GLM) functionality with a design matrix accounting for an interaction between SP and CT (referred to as SP × CT-DE analysis). We performed an ANOVA-like test for differences across all samples. Furthermore, in order to determine what differences existed between species for each CT, we performed interspecies pairwise DE comparisons in a similar manner between NPCs, neurons, and astrocytes (referred to as CT-DE analyses). We also used the GLM for these analyses but did not include more than 1 CT in these analyses in order to include genes that may be CT-specific. For all analyses, we used edgeR's quasi-likelihood *F*-test and considered gene expression significantly different at an FDR of <5%. Normalization of data in edgeR for DE analyses ensured that DE is not dependent on original number of cells. All Venn diagrams were created using the R package Vennerable.

### Categorical Enrichment Analyses

Uninformed pathway enrichment analyses were conducted using genes identified as differentially expressed from each DE comparison using g:Profiler (Raudvere et al. 2019) with their functional enrichment tool (g:GOSt). Categorical enrichment analyses for overrepresented (enriched) and underrepresented (conserved) processes were conducted on all genes identified as differentially expressed (FDR < 0.05%) between species for individual CTs. Enrichments with a Q-value of <0.05 were considered significant.

### GSEA

In order to investigate which metabolic pathways were enriched in a species’ CT, we used GSEA (Subramanian et al. 2005). We tested for enrichment of 23 a priori gene sets from the MSigDB (Liberzon et al. 2011) using the raw counts of the same set of genes used for the CT-DE pairwise comparisons. Gene sets were considered significantly enriched according to suggested thresholds (FDR < 25% and nominal *P*-value < 0.05) (Subramanian et al. 2005). Leading edge analyses determined a set of core-enriched genes that most significantly influenced the enrichment of the gene set per phenotype.

### Selection Analyses

In order to determine if genes exhibiting significant interspecies DE also had evidence of positive selection in their coding sequences, we used dN and dS nucleotide changes per gene for all genes expressed in iPSC-derived neural cells. These were obtained from Ensembl using BioMart (Kinsella et al. 2011). These precalculated dN and dS values were originally computed by Ensembl using codeml and yn00 of the PAML package to compute dN and dS scores for each species in comparison with human (Herrero et al. 2016). A rate of change was calculated for each gene (dN/dS), where a dN/dS > 1 is indicative of positive selection (Herrero et al. 2016). In order to determine if there was evidence for noncoding selection, we analyzed promoter regions of genes involved in aerobic glycolysis. These genes were selected by downloading gene lists from the MSigDB (Liberzon et al. 2011) for GO pathways involved in aerobic glycolysis (glycolysis, pyruvate conversion to lactate or acetyl-CoA, TCA cycle, electron transport chain, OXPHOS) and further subset to those that were expressed in at least 1 sample (*n* = 156). Signs of positive selection in noncoding regions adjacent to these genes were determined following the procedures outlined in Pizzollo et al. (2018) (Haygood et al. 2007; Pizzollo et al. 2018). Because these analyses are suited for nuclear-encoded genes, we excluded mitochondrial-encoded genes (*n* = 10). Rhesus macaque (*Macaca mulatta*) was used as an outgroup. After removing regions without sequences for all 3 species (human, chimpanzee, and rhesus macaque), we tested for positive selection in the human lineage of a total of 126 aerobic glycolysis genes.

### Network Schematic

We constructed a focal set of signaling pathways based upon HumanCyc (Romero et al. 2005) in order to contextualize our DE results in the framework of a network signaling, and this is the diagram of the major pathways involved in aerobic glycolysis (glycolysis, PPP, lactate conversion from pyruvate, and TCA cycle) shown in Fig. 5. For each enzyme in the pathway, 3 blocks indicate expression of this enzyme in each CT (left to right): NPCs, neurons, and astrocytes. Color indicates the level of expression (higher in human [red], higher in chimpanzee [blue], not expressed in this CT [grey]).

## Supplementary Material

evad239_Supplementary_Data

## Data Availability

Sequence data are available on the Short Read Archive, accession numbers PRJNA940438 and PRJNA940448.
