# Supplementary material for: Astrocytes Drive Divergent Metabolic Gene Expression in Humans and Chimpanzees
Source: Genome Biol Evol. 2023 Dec 30;16(1):evad239. doi: 10.1093/gbe/evad239 (PMC10829071; doi:10.1093/gbe/evad239)
Supplement: evad239_Supplementary_Data [file evad239_supplementary_data.zip › Zintel_Supplemental Material Legends.docx]

**SI TABLE LIST**

**SI Table 1 |** Sample, cell line, and sequencing information.

**SI Table 2 |** External RNA-Seq Metadata from Gene Expression Omnibus.

**SI Table 3 |** Interspecies differentially expressed (DE) genes.

**SI Table 4 |** Categorical enrichment analyses of DE genes.

**SI Table 5 |** Gene Set Enrichment Analysis (GSEA) Pathway enrichment of DE genes.

**SI Table 6 |** Leading edge analyses of genes in three significant GSEA pathways.

SI Table 7 | **|** Interspecies differentially expressed genes with evidence of coding selection.

SI Table 8 | Interspecies differentially expressed genes with evidence of non-coding selection.

**SI FIGURE LIST**

**SI Figure 1 |** Differentiation and maturation of a human and chimpanzee iPSC lines into neural cell types.

**SI Figure 2 |** MDS plots of all iPSC-derived samples with shape indicating cell line.

**SI Figure 3 |** MDS plots of individual cell types ( A & D – NPCs, B & E – neurons, C & F – astrocytes).

**SI Figure 4 |** MDS plots of all A & C) human and B & D) chimpanzee samples by cell type.

**SI Figure 5 |** Human and chimpanzee iPSC-derived neural cells resemble primary neural cell types and tissue regions more than non-neuronal tissues.

**SI Figure 6 |** Distribution of differentially expressed genes between species for each cell type.

**SI Figure 7 |** Overlap in interspecies CT-DE genes.

**SI Figure 8 |** GO Biological Process (BP) enrichments.

**SI Figure 9 |** GO Cellular Component (CC) enrichments.

**SI Figure 10 |** GO Molecular Function (MF) enrichments.

**SI Figure 11 |** Neurons and astrocytes exhibit contrasting interspecies differences in lactate and glucose transport.

**SI Figure 12** | Positive selection in the coding regions of genes expressed in iPSC-derived neural cells.
