# Supplementary material for: Astrocytes Drive Divergent Metabolic Gene Expression in Humans and Chimpanzees
Source: Genome Biol Evol. 2023 Dec 30;16(1):evad239. doi: 10.1093/gbe/evad239 (PMC10829071; doi:10.1093/gbe/evad239)
Supplement: evad239_Supplementary_Data [file evad239_supplementary_data.zip › All_SI_Figures_combined.pdf]

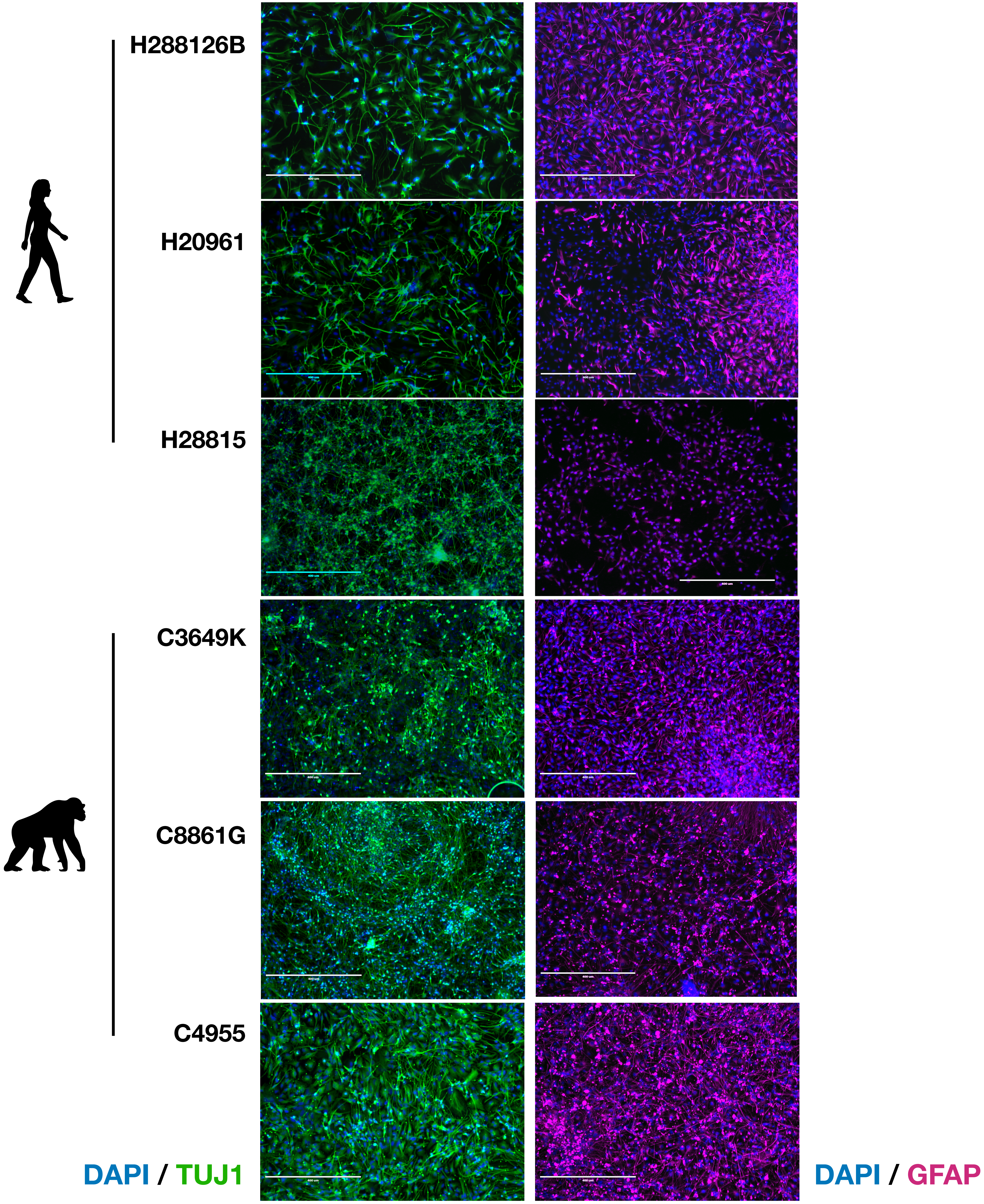
H288126B
H20961
H28815
C3649K
C8861G
C4955
DAPI / TUJ1
DAPI / GFAP

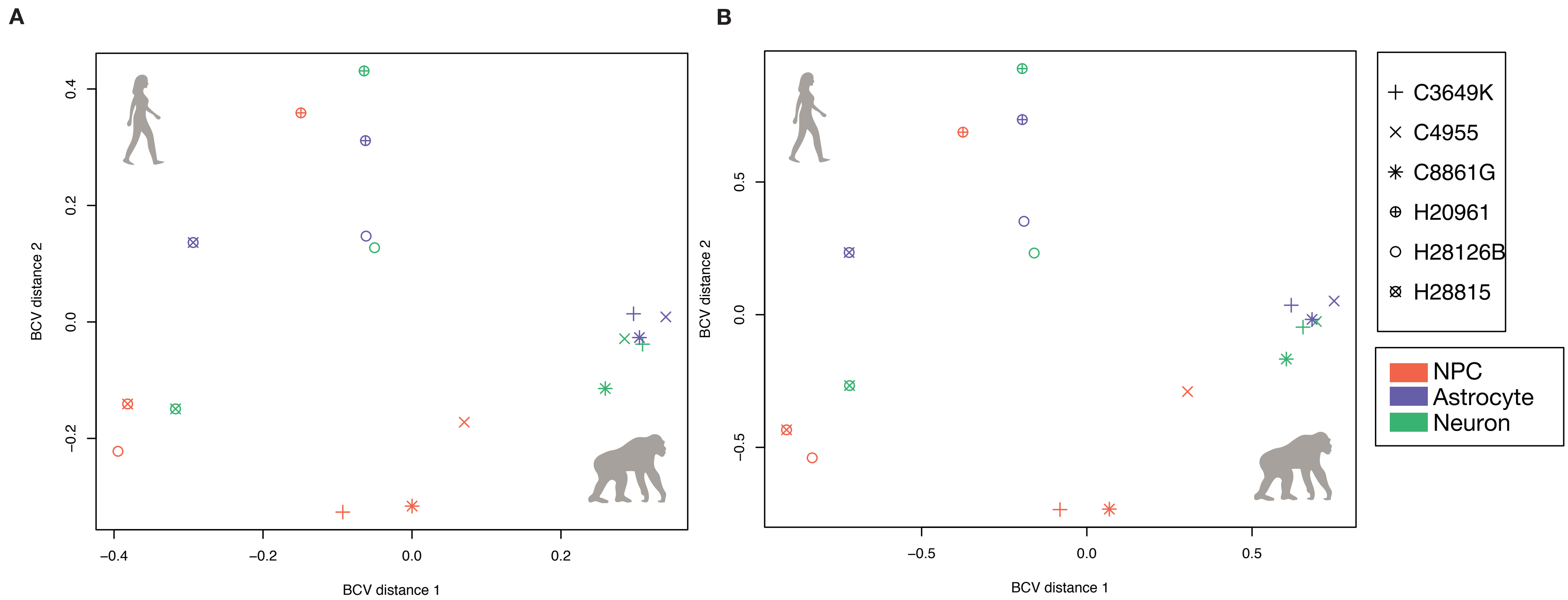
A
B
BCV distance 1
BCV distance 2
C3649K
C4955
C8861G
H20961
H28126B
H28815
NPC
Astrocyte
Neuron

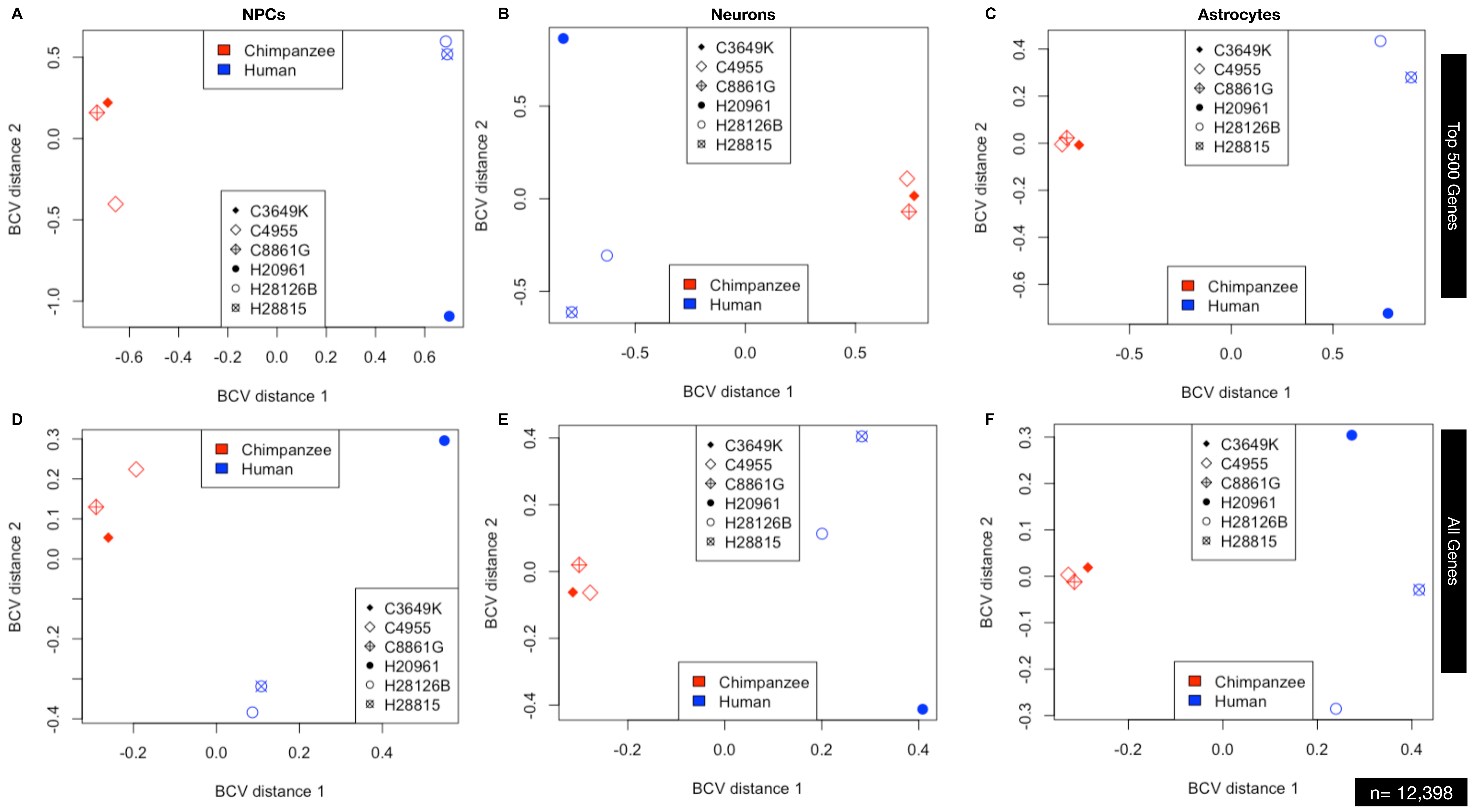
A
NPCs
B
Neurons
C
Astrocytes
D
E
F
Chimpanzee
Human
C3649K
C4955
C8861G
H20961
H28126B
H28815
BCV distance 1
BCV distance 2
Top 500 Genes
All Genes
n= 12,398

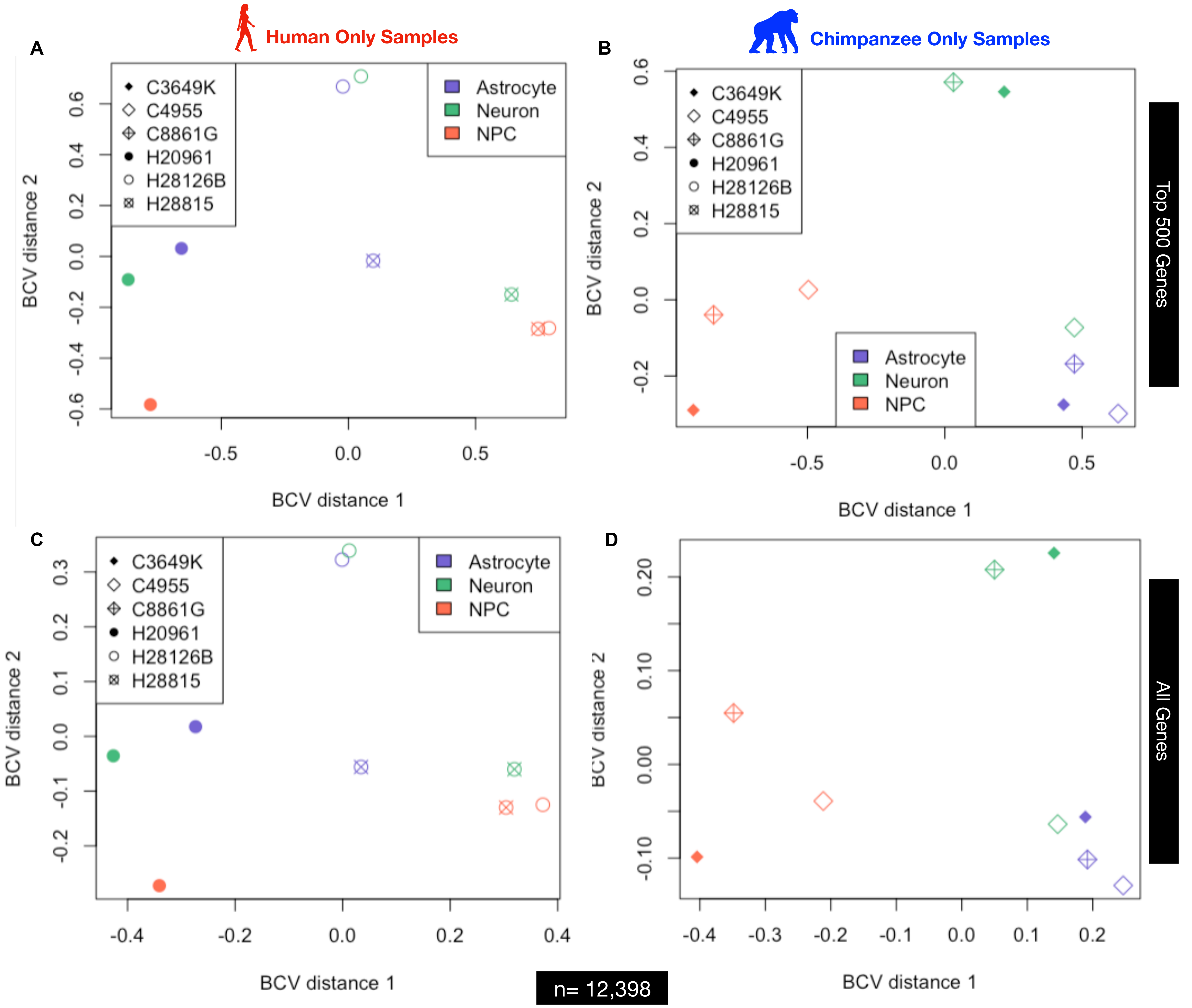
Human Only Samples
Chimpanzee Only Samples
A
B
C
D
C3649K
C4955
C8861G
H20961
H28126B
H28815
Astrocyte
Neuron
NPC
BCV distance 1
BCV distance 2
Top 500 Genes
All Genes
n= 12,398

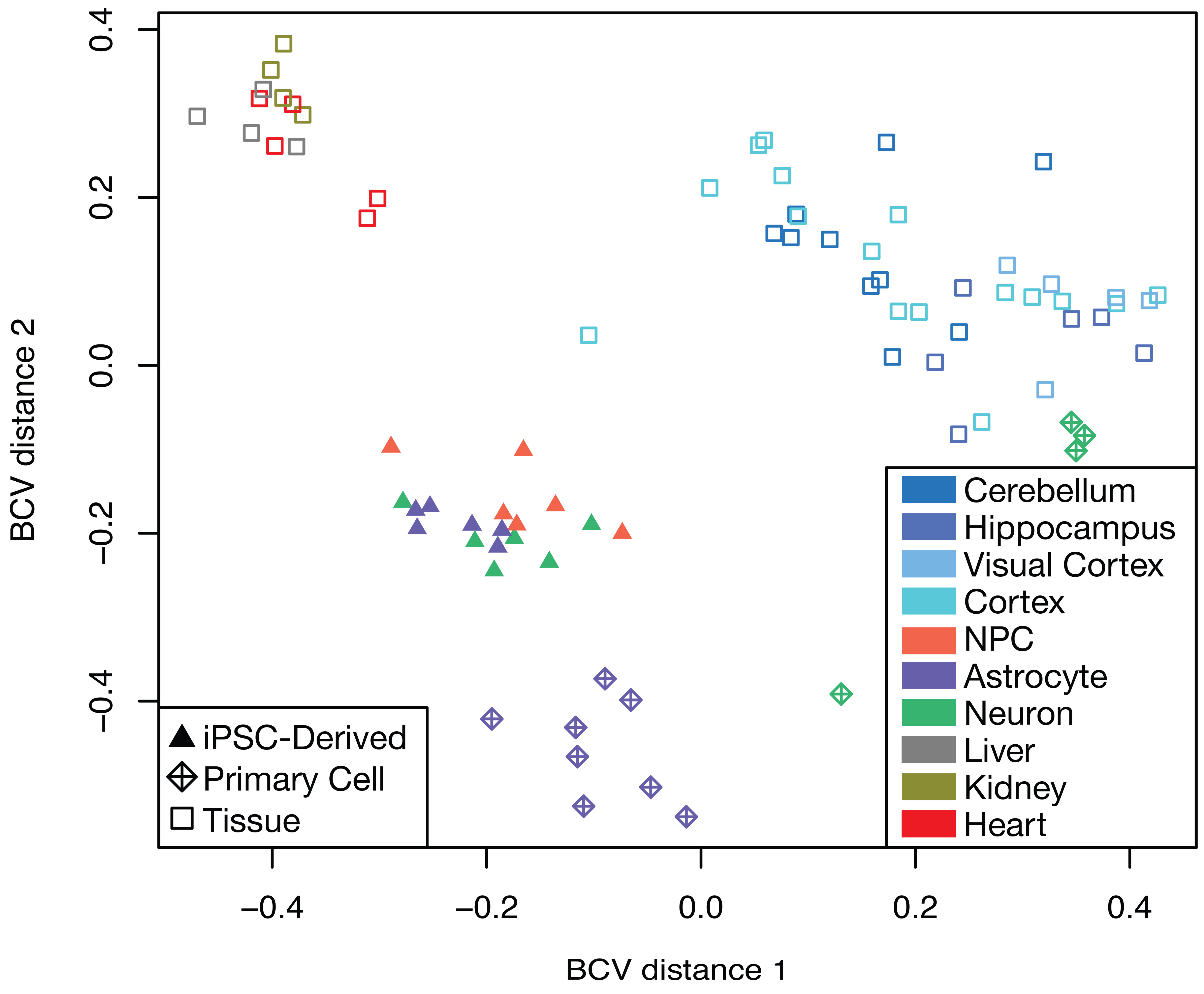

BCV distance 2
BCV distance 1
0.4
0.2
0.0
−0.2
−0.4
−0.4
−0.2
0.0
0.2
0.4
iPSC-Derived
Primary Cell
Tissue
Cerebellum
Hippocampus
Visual Cortex
Cortex
NPC
Astrocyte
Neuron
Liver
Kidney
Heart

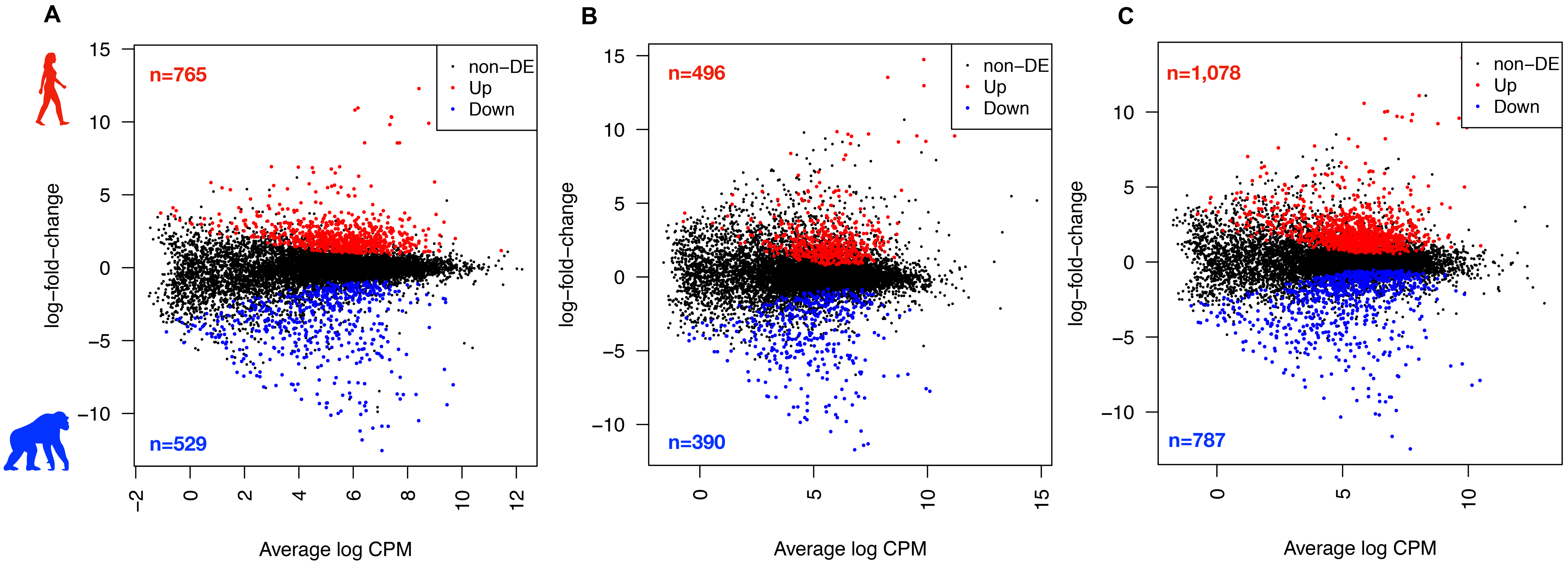

DE Total: 1,294
Total genes expr. in all NPCs: 12,481
Genes after filtering CPM > 1: 11,772
% DE:
Ratio (H/C): 1.45

DE Total: 886
Total genes expr. in all Neurons: 12,451
Genes after filtering CPM > 1: 12,046
% DE:
Ratio (H/C): 1.27

DE Total: 1,865
Total genes expr. in all Astrocytes: 12,302
Genes after filtering CPM > 1: 11,854
% DE:
Ratio (H/C): 1.34

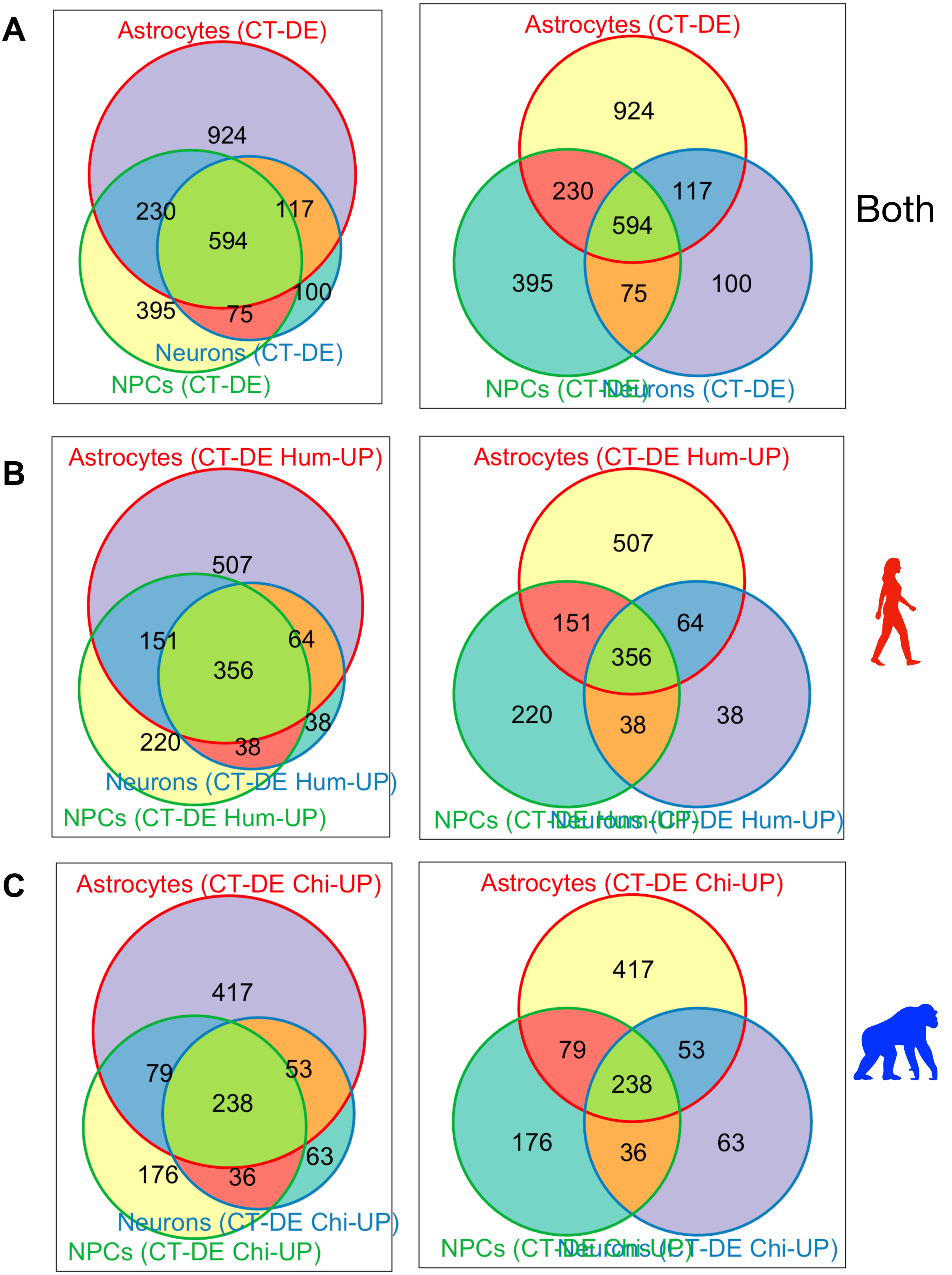
A
Astrocytes (CT-DE)
924
230
117
594
395
75
100
Neurons (CT-DE)
NPCs (CT-DE)
Astrocytes (CT-DE)
924
230
117
594
395
75
100
NPCs (CT-DE)
Neurons (CT-DE)
Both
B
Astrocytes (CT-DE Hum-UP)
507
151
64
356
220
38
38
Neurons (CT-DE Hum-UP)
NPCs (CT-DE Hum-UP)
Astrocytes (CT-DE Hum-UP)
507
151
64
356
220
38
38
NPCs (CT-DE Hum-UP)
Neurons (CT-DE Hum-UP)
C
Astrocytes (CT-DE Chi-UP)
417
79
53
238
176
36
63
Neurons (CT-DE Chi-UP)
NPCs (CT-DE Chi-UP)
Astrocytes (CT-DE Chi-UP)
417
79
53
238
176
36
63
NPCs (CT-DE Chi-UP)
Neurons (CT-DE Chi-UP)

A

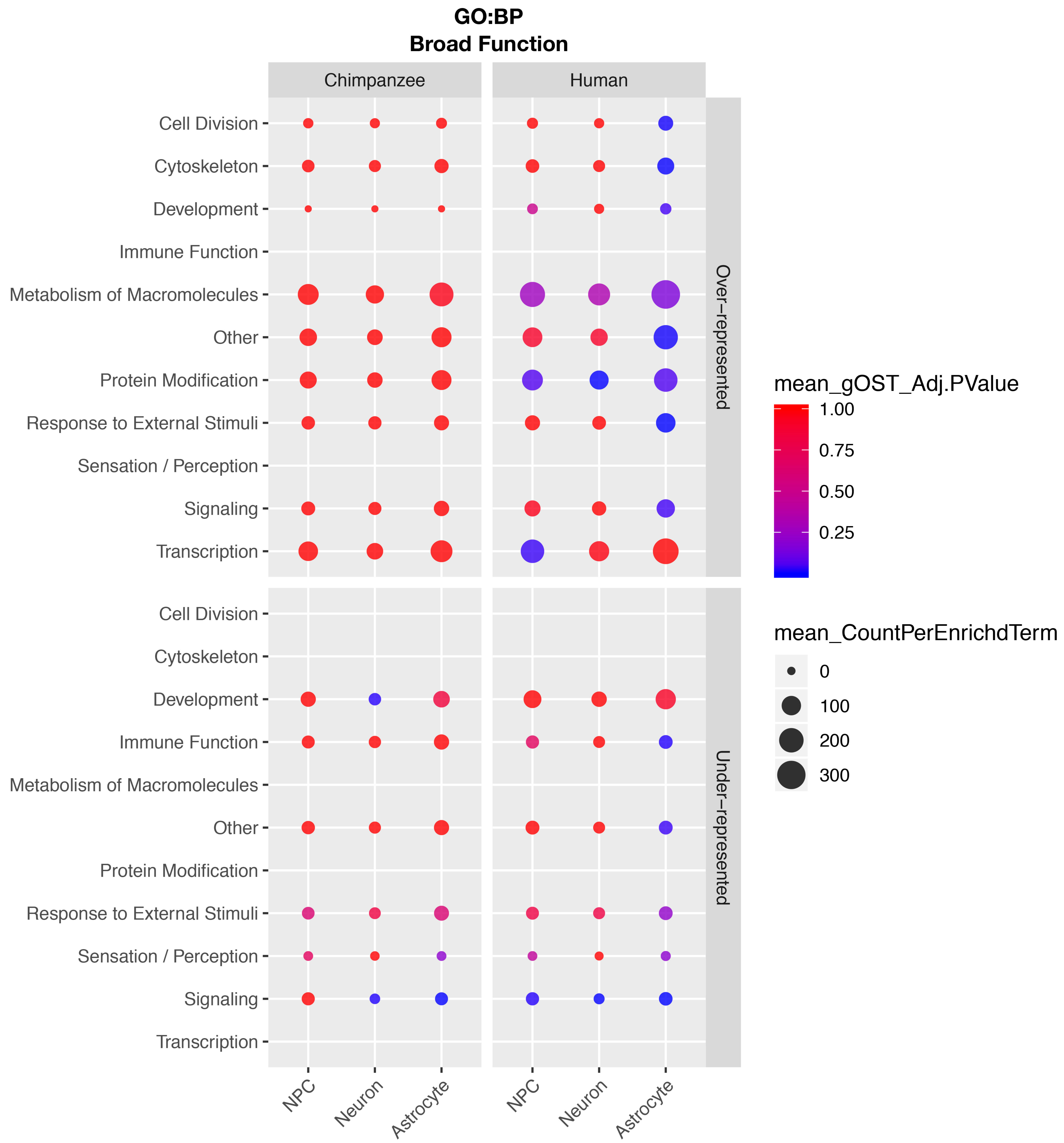

GO:BP
Broad Function
Chimpanzee
Human
Over−represented
Under−represented
Cell Division
Cytoskeleton
Development
Immune Function
Metabolism of Macromolecules
Other
Protein Modification
Response to External Stimuli
Sensation / Perception
Signaling
Transcription
NPC
Neuron
Astrocyte
mean_gOST_Adj.PValue
1.00
0.75
0.50
0.25
mean_CountPerEnrichdTerm
0
100
200
300

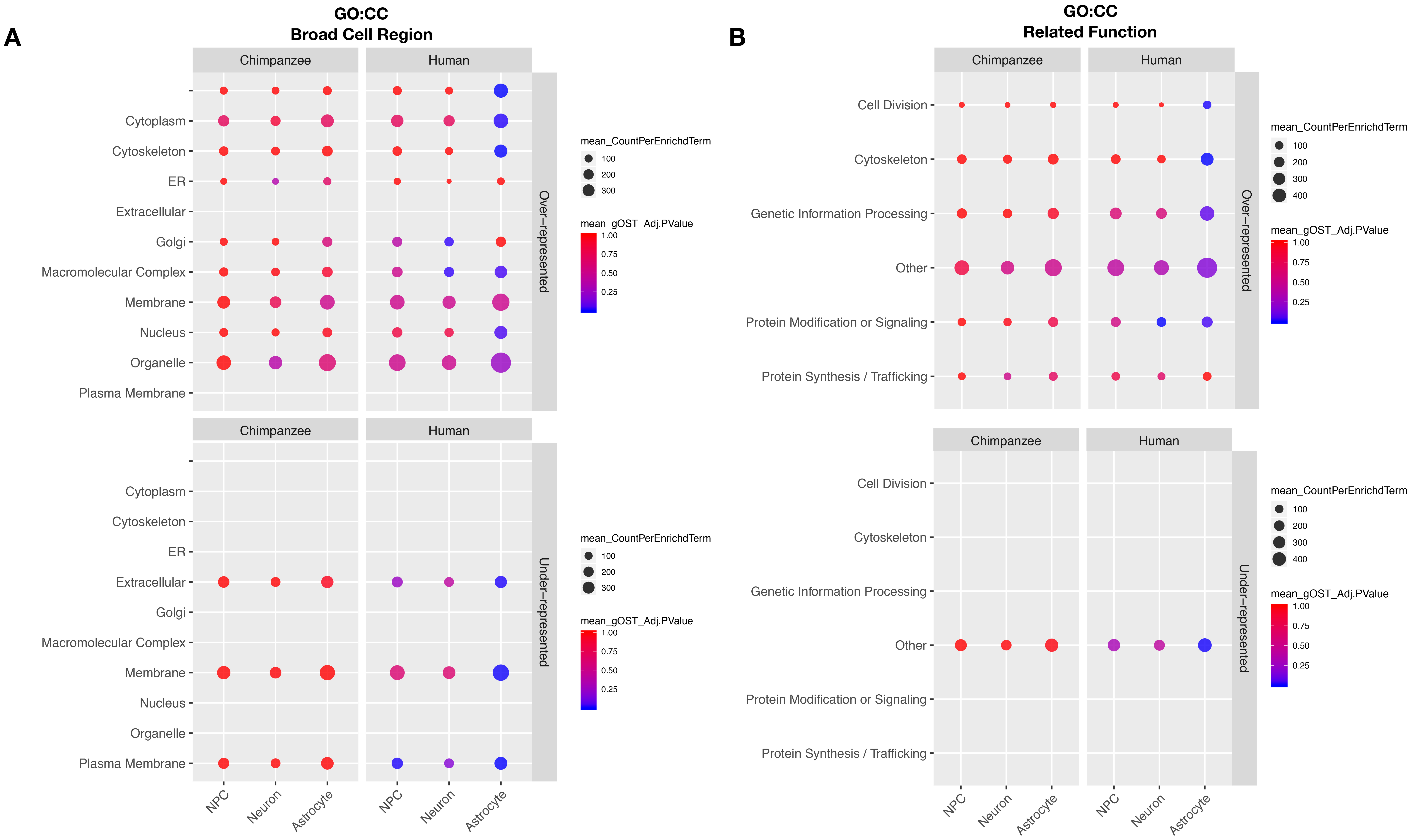
A
GO:CC
Broad Cell Region
Chimpanzee
Human
Cytoplasm
Cytoskeleton
ER
Extracellular
Golgi
Macromolecular Complex
Membrane
Nucleus
Organelle
Plasma Membrane
Over−represented
Under−represented
NPC
Neuron
Astrocyte
mean_CountPerEnrichdTerm
100
200
300
mean_gOST_Adj.PValue
1.00
0.75
0.50
0.25
B
GO:CC
Related Function
Cell Division
Cytoskeleton
Genetic Information Processing
Other
Protein Modification or Signaling
Protein Synthesis / Trafficking
400

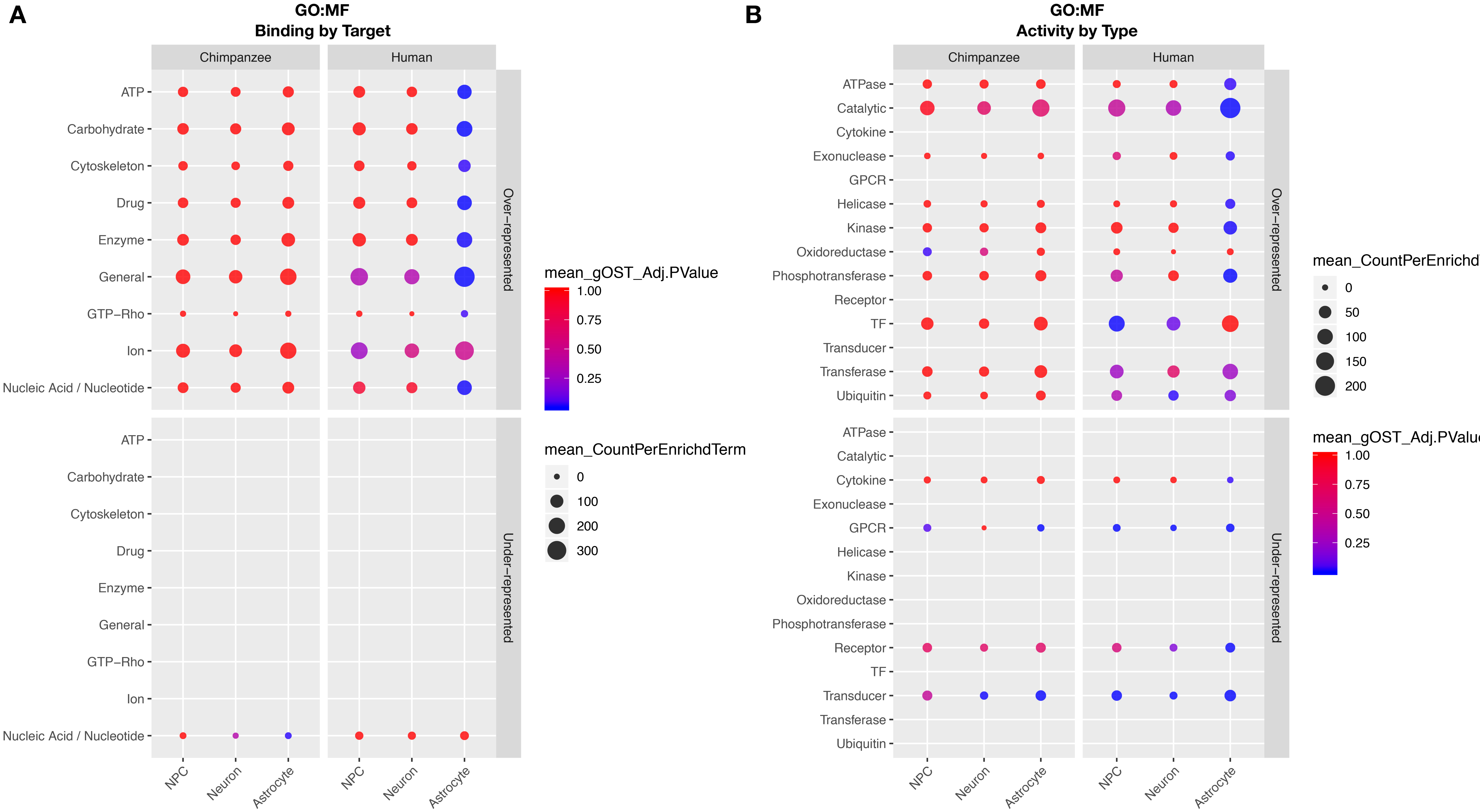

A
GO:MF
Binding by Target
Chimpanzee
Human
ATP
Carbohydrate
Cytoskeleton
Drug
Enzyme
General
GTP–Rho
Ion
Nucleic Acid / Nucleotide
Over–represented
Under–represented
NPC
Neuron
Astrocyte
mean_gOST_Adj.PValue
1.00
0.75
0.50
0.25
mean_CountPerEnrichdTerm
0
100
200
300
B
GO:MF
Activity by Type
ATPase
Catalytic
Cytokine
Exonuclease
GPCR
Helicase
Kinase
Oxidoreductase
Phosphotransferase
Receptor
TF
Transducer
Transferase
Ubiquitin
mean_CountPerEnrichd
0
50
100
150
200
mean_gOST_Adj.PValu

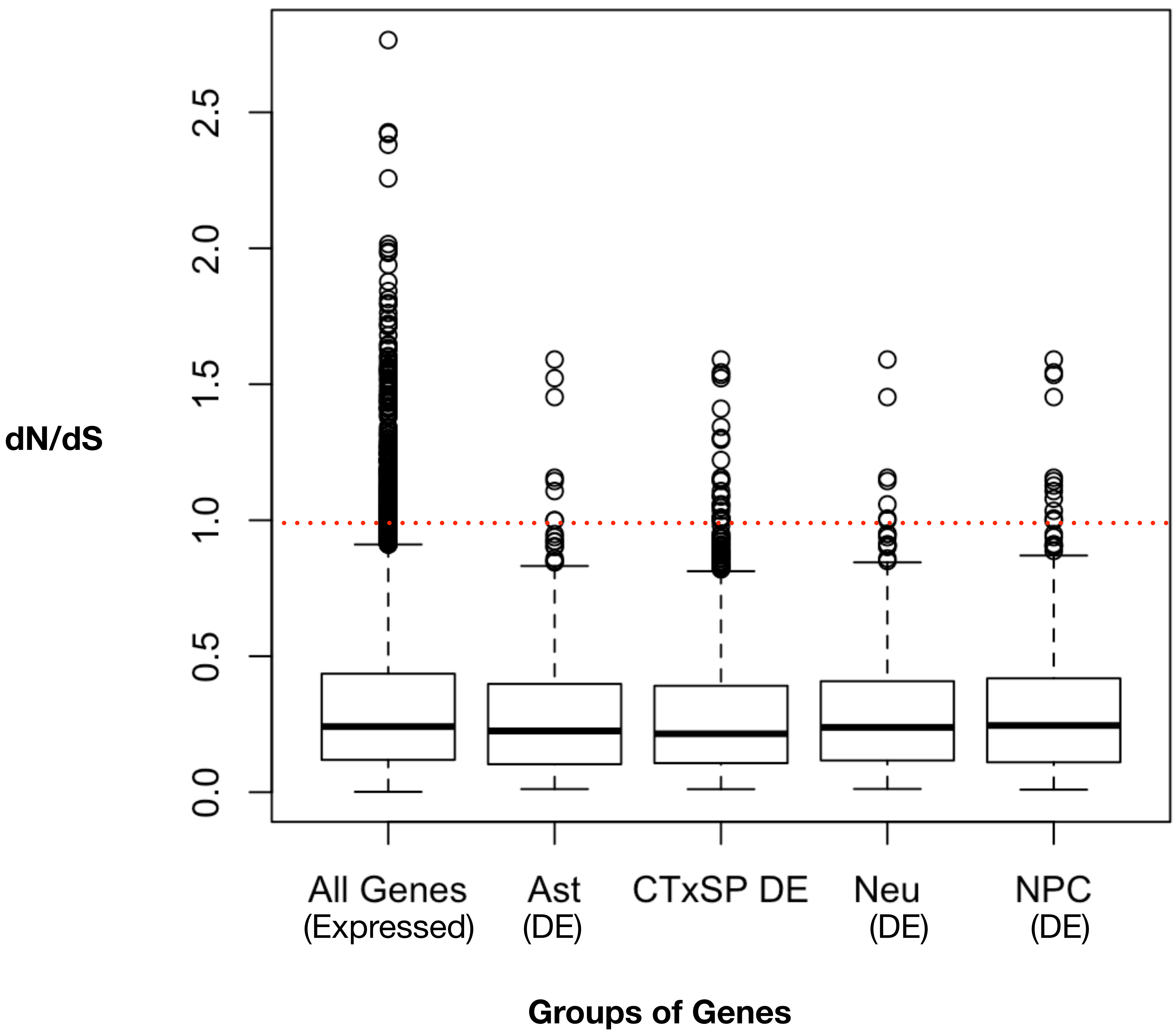
dN/dS
0.0
0.5
1.0
1.5
2.0
2.5
All Genes (Expressed)
Ast (DE)
CTxSP DE
Neu (DE)
NPC (DE)
Groups of Genes

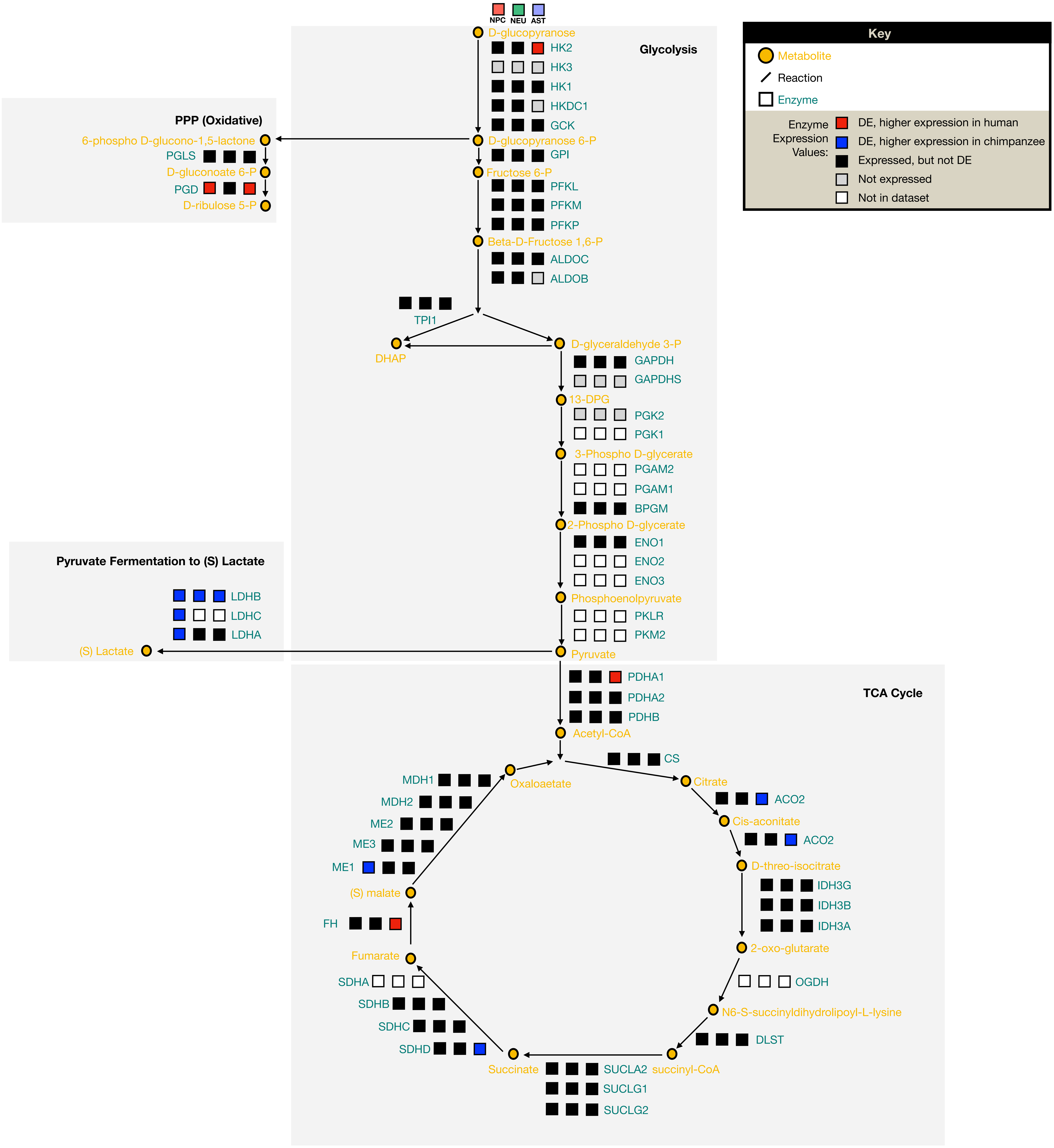
NPC
NEU
AST
Glycolysis
D-glucopyranose
HK2
HK3
HK1
HKDC1
GCK
D-glucopyranose 6-P
GPI
Fructose 6-P
PFKL
PFKM
PFKP
Beta-D-Fructose 1,6-P
ALDOC
ALDOB
TPI1
DHAP
D-glyceraldehyde 3-P
GAPDH
GAPDHS
13-DPG
PGK2
PGK1
3-Phospho D-glycerate
PGAM2
PGAM1
BPGM
2-Phospho D-glycerate
ENO1
ENO2
ENO3
Phosphoenolpyruvate
PKLR
PKM2
Pyruvate
PPP (Oxidative)
6-phospho D-glucono-1,5-lactone
PGLS
D-gluconoate 6-P
PGD
D-ribulose 5-P
Pyruvate Fermentation to (S) Lactate
LDHB
LDHC
LDHA
(S) Lactate
Key
Metabolite
Reaction
Enzyme
Enzyme Expression Values:
DE, higher expression in human
DE, higher expression in chimpanzee
Expressed, but not DE
Not expressed
Not in dataset
TCA Cycle
PDHA1
PDHA2
PDHB
Acetyl-CoA
CS
Citrate
ACO2
Cis-aconitate
ACO2
D-threo-isocitrate
IDH3G
IDH3B
IDH3A
2-oxo-glutarate
OGDH
N6-S-succinyldihydrolipoyl-L-lysine
DLST
succinyl-CoA
SUCLA2
SUCLG1
SUCLG2
Succinate
SDHA
SDHB
SDHC
SDHD
Fumarate
FH
(S) malate
ME1
ME3
ME2
MDH2
MDH1
Oxaloacetate
